# PGC7 promotes tumor oncogenic dedifferentiation through remodeling DNA methylation pattern for key developmental transcription factors

**DOI:** 10.1038/s41418-020-00726-3

**Published:** 2021-01-26

**Authors:** Qian Yan, Yu Zhang, Xiaona Fang, Beilei Liu, Tin Lok Wong, Lanqi Gong, Shan Liu, Dandan Yu, Ming Liu, Lingxi Jiang, Xia Wang, Ting Wei, Yongxu Jia, Lei Li, Liangzhan Sun, Ying Tang, Ningning Zhou, Yun-Fei Yuan, Yan Li, Stephanie Ma, Xin-Yuan Guan

**Affiliations:** 1 Research Center of Medical Sciences, Guangdong Provincial People’s Hospital, Guangdong Academy of Medical Sciences, Guangzhou, China; 2 Department of Clinical Oncology, The University of Hong Kong-Shenzhen Hospital, Hong Kong, China; 3 Department of Clinical Oncology, The University of Hong Kong, Hong Kong, China; 4 State Key Laboratory for Liver Research, The University of Hong Kong, Hong Kong, China; 5 School of Biomedical Sciences, University of Hong Kong, Pokfulam, Hong Kong, China; 6 Key Laboratory of Protein Modification and Degradation, School of Basic Medical Sciences, Affiliated Cancer Hospital & Institute of Guangzhou Medical University, Guangzhou Medical University, Guangzhou, China; 7 Department of Surgery, Ruijin Hospital, Shanghai Jiao Tong University School of Medicine, 200025 Shanghai, China; 8 Department of Oncology, Zhujiang Hospital, Southern Medical University, Guangzhou, China; 9 Department of Clinical Oncology, The First Affiliated Hospital, Zhengzhou University, Zhengzhou, China; 10 Gastro-Intestinal Cancer Center of Henan Province, Zhengzhou, China; 11 State Key Laboratory of Oncology in Southern China, Sun Yat-sen University Cancer Center, 510060 Guangzhou, China

**Keywords:** Genetics research, Stem-cell research

## Abstract

Poorly differentiated tumors usually exhibit phenotypes similar to that of their developmental precursor cells. Tumor cells that acquire the lineage progenitor cells feature usually exploit developmental signaling to potentiate cancer progression. However, the underlying molecular events remain elusive. In this study, based on analysis of an in vitro hepatocyte differentiation model, the maternal factor PGC7 (also known as DPPA3, STELLA) was found closely associated with liver development and tumor differentiation in hepatocellular carcinoma (HCC). Expression of PGC7 decreased during hepatocyte maturation and increased progressively from well-differentiated HCCs to poorly differentiated HCCs. Whole-genome methylation sequencing found that PGC7 could induce promoter demethylation of genes related to development. Pathway-based network analysis indicated that downstream targets of PGC7 might form networks associated with developmental transcription factor activation. Overexpression of PGC7 conferred progenitor-like features of HCC cells both in vitro and in vivo. Mechanism studies revealed that PGC7 could impede nuclear translocation of UHRF1, and thus facilitate promoter demethylation of GLI1 and MYCN, both of which are important regulators of HCC self-renewal and differentiation. Depletion or inhibition of GLI1 effectively downregulated MYCN, abolished the effect of PGC7, and sensitized HCC cells to sorafenib treatment. In addition, we found a significant correlation of PGC7 with GLI1/MYCN and lineage differentiation markers in clinical HCC patients. PGC7 expression might drive HCC toward a “dedifferentiated” progenitor lineage through facilitating promoter demethylation of key developmental transcription factors; further inhibition of PGC7/GLI1/MYCN might reverse poorly differentiated HCCs and provide novel therapeutic strategies.

## Introduction

It has been well-studied that cancer cells usually exhibited phenotypic similarity with their lineage precursor cells during development [1]. The gene expression signature during normal organ development was reactivated in tumor cells, which substantially contribute to tumor malignant transformation [2]. Hepatocellular carcinoma (HCC) is one of the frequently diagnosed cancers worldwide with an inferior prognosis [3]. Poorly differentiated HCCs retaining the developing signaling of their liver precursors are usually highly aggressive with poor clinical outcome [4]. For example, HCCs which regain the expression of bipotential hepatic progenitor markers such as cytokeratin 7 (CK7) or CK19 is predicted to have an extremely poor prognosis [5]. This evidence convinced us that the mechanisms governing tumor cells lineage reversion should be of critical importance during HCC malignant transformation.

Recently we established an in vitro hepatocyte differentiation model to mimic liver development and HCC progression [6]. The differentiation process comprises four stages—embryonic stem cell, endoderm, liver progenitor cell, and premature hepatocyte stages. Combining clinical liver cancer transcriptomic data, we analyzed different gene expression patterns and mainly focus on actively expressed genes derived from liver progenitors and tumor tissues. Based on the selection criteria, the maternal factor PGC7 arouse our interest since it ranks at the top of the list, and has a unique expression pattern strongly associated with tumor differentiation and poor clinical outcomes.

PGC7/DPPA3 belongs to the developmental pluripotency-associated protein (DPPA) family and was found frequently expressed in pluripotent cells [7]. PGC7 protects DNA methylation pattern in early embryogenesis [8] by binding to either dimethylated histone H3K9 (H3K9me2) [9] or TET2/TET3 [10]. In addition, PGC7 was found to safeguard oocyte methylome by interacting with UHRF1 and DNMT1 [11]. Overexpression of PGC7 enhanced the metastatic ability of melanoma cells coupled with global DNA demethylation [12]. Expression of PGC7 was also found indispensable for the generation of induced pluripotent stem cells (iPSCs) [13]. All of the evidence indicated that PGC7 might be critical in lineage-related tumorigenesis through remodeling DNA methylation patterns.

In the present study, we established a close association of PGC7 expression with liver development and HCC lineage reversion. Overexpression of PGC7 conferred lineage progenitor-like features of tumor cells both in vitro and in vivo. Mechanism studies revealed that PGC7 could facilitate a developmental pluripotency-related transcriptional program through remodeling DNA methylation patterns at key transcription factors. We proposed a novel mechanism in which ectopic expression of PGC7 could promote HCC oncogenic dedifferentiation and maintain an epigenetic status suitable for liver progenitor cells, which further contributed to metastasis and poor prognosis of HCC.

## Methods

### Cell Lines and HCC clinical specimens

107 pairs of primary HCC specimens and their adjacent non-tumor tissues used for TMA were collected from patients who underwent hepatectomy from HCC at Sun Yat-Sen University Cancer Center (Guangzhou, China). All HCC patients gave written informed consent on the use of clinical specimens for medical research. The samples used in this study were approved by the Committees for the Ethical Review of Research Involving Human Subjects at the Sun Yat-Sen University Cancer Center. Histological examination was carried out by pathologists. Tumors grades were determined according to the American Joint Committee on Cancer (AJCC)/Union for International Cancer Control (UICC) grading system. Investigators were blinded for patient histopathological diagnosis when counting PGC7 positive staining. Human immortalized liver cell lines MIHA and HCC cell lines MHCC-97H, SNU378, H2P, Huh7, Hep3B, SNU449, H2M, SNU475, PLC-8024 were used in this study and tested for mycoplasma contamination. STR DNA profiling analysis was conducted for cell lines authentication. Cells were maintained in high-glucose Dulbecco’s modified Eagle medium (Gibco BRL, Grand Island, NY) supplemented with 10% fetal bovine serum (Gibco BRL) and 1% penicillin/streptomycin. Cells were incubated at 37 °C in a humidified incubator containing 5% CO_2_.

### Establishment of an in vitro hepatocyte differentiation model

The derivation of hESCs and their use for research was approved by the ethical committee of the CITIC-Xiangya Reproductive & Genetic Hospital. In brief, hESCs were cultured at a density of about 2500 cells/cm^2^ on a feeder layer of mitotically inactivated human embryonic fibroblasts. The culturing medium of hESCs was KO/DMEM medium (Life Technologies) supplemented with 15% knockout serum replacement (SR), 2mM l-glutamine, 2 mM nonessential amino acids, 0.1 mM β-mercaptoethanol, and 4 ng/ml of basic fibroblast growth factor. hESCs were passaged when the confluence was between 50 and 70%, then cultured in RPMI-1640 supplemented with 100 ng/ml activin A (R&D Systems) and 25 ng/ml Wnt3a (R&D Systems) for 3 days until definitive endoderm was specified. To induce hepatic endoderm, cells were growing in KO/DMEM medium supplemented with 25 nm/ml keratinocyte growth factor (R&D Systems) and 2% FBS (Gibco) for two days, followed by culturing in the KO/DMEM medium containing 20% SR, 1% nonessential amino acids, 1 mM glutamine, 0.1 mM 2-mercaptoethanol and 1% DMSO for 4–7 days. To generate mature hepatocytes, cells were cultured in the mature medium consisting of 10% FBS, 10 ng/ml hepatocyte growth factor (R&D Systems), 20 ng/ml Oncostatin M (R&D Systems), and 0.5 μM dexamethasone (R&D Systems) for 7 more days.

### In vitro and in vivo functional assays

The in vitro self-renewal capability was assessed by sphere formation assay, drug resistance assay, and ALDH activity assay in cell lines and patient-derived organoids. In vivo tumorigenic ability was investigated in a xenograft mouse model. A detailed description of the HCC organoid establishment and mice model could be found in the Supplementary Materials and Methods section.

### Methylation sequencing analysis

The platform and normalization methods of whole-genome methylation sequencing were introduced in the supplementary materials and methods section. Sequences of primers used in Qrt-PCR and bisulfite genomic sequencing were listed in Supplementary Table 1.

### Statistics

Statistical analysis was performed using SPSS (version 16.0; SPSS, Chicago, IL). Pearson *χ*^2^ test was used to analyze the association of PGC7 expression with clinicopathologic parameters. Kaplan–Meier plot and log-rank test were used for survival analysis. Independent Student *t*-test was used for most studies as indicated in the figure legends. The Levene or Brown–Forsynth test was used to compare the variance between the two groups. For limiting dilution assay, the tumor-initiating frequency and statistics were calculated using ELDA online platform (http://bioinf.wehi.edu.au/software/elda/). The data are presented as the mean ± standard deviation of three independent experiments. The *P*-values were denoted as *P < 0.05, **P < 0.01, ***P < 0.001 in all figures.

## Results

### The distinct gene expression pattern is identified in an in vitro hepatocyte differentiation model

**Fig. 1:**
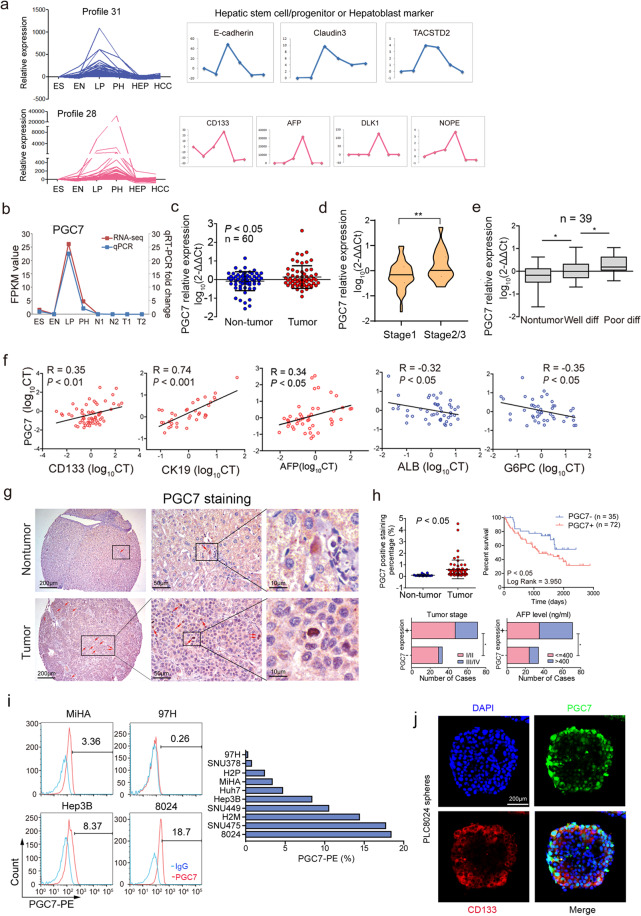
Association of PGC7 expression with liver development and HCC oncogenic dedifferentiation. **a** STEM (Short Time-series Expression Miner)-based trend analysis revealed two typical gene expression patterns in the model of in vitro hepatocyte differentiation. ES embryonic stem cell, EN endoderm, LP liver progenitor cell, PH premature hepatocytes, HEP average value of two cases of non-tumor tissues, HCC average value of two cases of tumor tissues. **b** PGC7 expression pattern was verified in the model of in vitro hepatocyte differentiation by qRT-PCR. Data were shown as the mean of three independent experiments. N1, N2, two cases of non-tumor specimens; T1, T2, two cases of HCC specimens. **c** PGC7 expression level was detected in 60 pairs of HCC samples by qRT-PCR. A paired *t*-test was used for statistical analysis. **d** PGC7 expression was detected in HCC clinical samples divided by tumor stage. Early-stage—stage1; advanced stage—stage 2 or 3; Student’s *t*-test was used for statistical analysis, **P < 0.01. **e** Expression levels of PGC7 were elevated in poor differentiated HCC (Poor diff) compared with well-differentiated HCC (Well diff) and non-tumor tissues by qRT-PCR. Student’s *t*-test was used for statistical analysis, *P < 0.05. **f** The association of PGC7 with CD133 (*n* = 61), CK19 (*n* = 36), AFP (*n* = 45), ALB (*n* = 46), and G6PC (*n* = 44) in HCC clinical specimens were detected by qRT-PCR. Pearson coefficient *R* was used to denote the expression correlation. **g** Representative IHC staining of PGC7 in non-tumor tissues and HCC clinical specimens. **h** Quantification of the PGC7 positive cells in Tumor and para-tumor tissues (upper left); Kaplan–Meier survival analysis showed HCC patients with PGC7 positive staining (*n* = 72) had worse survival outcome compared with patients without detectable PGC7 expression (*n* = 35) (upper right); Expression of PGC7 significantly associated with advanced tumor stage and relatively high AFP level (Fisher’s Exact Test, *P < 0.05, lower panel). **i** Percentage of PGC7 positive cells detected by FACS in immortalized liver and HCC cell lines. Data were shown as the mean of three independent experiments. **j** Representative dual-color immunofluorescence analysis of cryosectioned spheroids generated from PLC-8024 cells showing the colocalization of PGC7 (green) and CD133 (red). Three independent experiments were conducted.

Recently we established a novel in vitro hepatocyte differentiation model, in which human embryonic stem cells (hESCs) were differentiated into hepatocytes with defined culture medium and growth factors. Transcriptomic data were collected for the four developmental stages—embryonic stem cell, endoderm, liver progenitor, and premature hepatocyte, together with two paired HCC clinical tissues. STEM-based trend analysis was performed and as shown in Supplementary Fig. 1a, 13 gene expression patterns were significantly enriched. The genes in profile 31 and 28 shared a similar pattern, in which they have a maximal expression in liver progenitor stage (profile 31) or premature hepatocytes (profile 28), and then markedly decrease or are entirely silenced in adult hepatocytes. Interestingly, hepatic stem cell/progenitor or hepatoblast markers reported by previous studies including E-cadherin [14], Claudin3 [15], TACSTD2 [16] were identified in profile 31, while CD133 [17], AFP [18], DLK1 [19], and NOPE [20] were found in profile 28 (Fig. 1a). Bioinformatics-aided pathway and gene ontology analysis revealed that this cluster of genes was closely associated with cellular development, cell differentiation, and signaling regulating pluripotency of stem cells (Supplementary Fig. 1b), among which PGC7 arouse our interest as it ranks at the top of the list.

### PGC7 is highly expressed in pluripotent cells and poorly differentiated tumor tissues

The expression pattern of PGC7 in the hepatocyte differentiation model was further confirmed by qRT-PCR. PGC7 reached its peak expression in the liver progenitor stage and then rapidly dropped after differentiation (Fig. 1b). We analyzed PGC7 expression in Christodoulou’s cohort from the GEO database (GSE27087) and found similar to GLI1, SOX2, NANOG, and OCT3/4, PGC7 expression decreased upon differentiation from iPSCs or ES cells to endoderm (Supplementary Fig. 1c), indicating its role in pluripotency maintenance. The expression of PGC7 was detected by qRT-PCR in 60 pairs of HCC specimens, and it was upregulated significantly in advanced-stage tumors, compared with early-stage tumors and normal liver tissues (Fig. 1c, d). As poorly differentiated tumors always preserve the molecular signature of their developmental precursor cells, we firstly examined PGC7 expression in our HCC cohort divided by the differentiation stage. The result revealed PGC7 was evidently upregulated in poorly differentiated tumor tissues, compared with well-differentiated tumor and normal liver tissues (Fig. 1e). Molecular markers that are activated during liver development or regeneration such as CD133, CK19, AFP, SOX9, EpCAM, CD90, CD105 as well as hepatocyte terminal differentiation markers G6PC and ALB were detected by qRT-PCR in clinical samples. The association study exhibited that PGC7 expression was positively correlated with the developmental markers and negatively correlated with the differentiation markers mentioned above (Fig. 1f and Supplementary Fig. 1d). All of this evidence has linked PGC7 with liver development and HCC lineage reversion.

### PGC7 positive cells are markedly associated with poor clinical outcome

To examine the expression and distribution of PGC7 in clinical specimens, tissue microarray (TMA) comprising 107 pairs of HCC tissues and their non-tumor counterparts were used to perform IHC staining. PGC7 expression was detected in about 67.2% (72/107) HCC specimens but almost absent in the majority of normal liver tissues, with only 14 (13.1%) cases showed positive staining. PGC7 was strongly expressed in 0.08 to 4.55% of cancer cells scattered among the tumor tissue. Nevertheless, in normal liver tissue, the percentage of positive cells was around 0.025 to 0.3% (Fig. 1g, h). The clinical pathologic study revealed PGC7 positive staining correlates with poor survival outcome in HCC patients and was significantly associated with advanced tumor stage (Pearson *χ*^2^ test, *P* < 0.05) and relatively high AFP level (Pearson *χ*^2^ test, *P* < 0.05) (Fig. 1h). Co-staining of PGC7 and hepatic progenitor marker CK19 in HCC samples revealed that the majority of PGC7 positive cells were co-expressed with CK19, indicating the marking of undifferentiated cells by PGC7 (Supplementary Fig. 1e).

Furthermore, the frequency of PGC7 positive cells was detected by flow cytometry in 9 HCC cell lines and 1 immortalized liver cell lines. The proportion of PGC7 positive cells ranged from 0.26 to 18.7% (Fig. 1i). Also, we found PGC7 was strongly expressed in liver cancer stem cells (CSCs) enriched from CD133^+^ populations of Huh7 and Hep3B cells (Supplementary Fig. 1f) as well as tumor spheroids (Supplementary Fig. 1g). Moreover, cryosectioned spheroids produced from PLC-8024 or SNU475 were double-stained with antibodies specific to PGC7 and CD133. We noticed that most of the PGC7 positive cells were co-expressed with CD133 (Fig. 1j and Supplementary Fig. 1h). Collectively, PGC7 may represent and mark a specific population of cancer cells with lineage reversed progenitor features and poor differentiation status, resulting in cancer progression.

### PGC7 promotes HCC oncogenic dedifferentiation both in vitro and in vivo

**Fig. 2:**
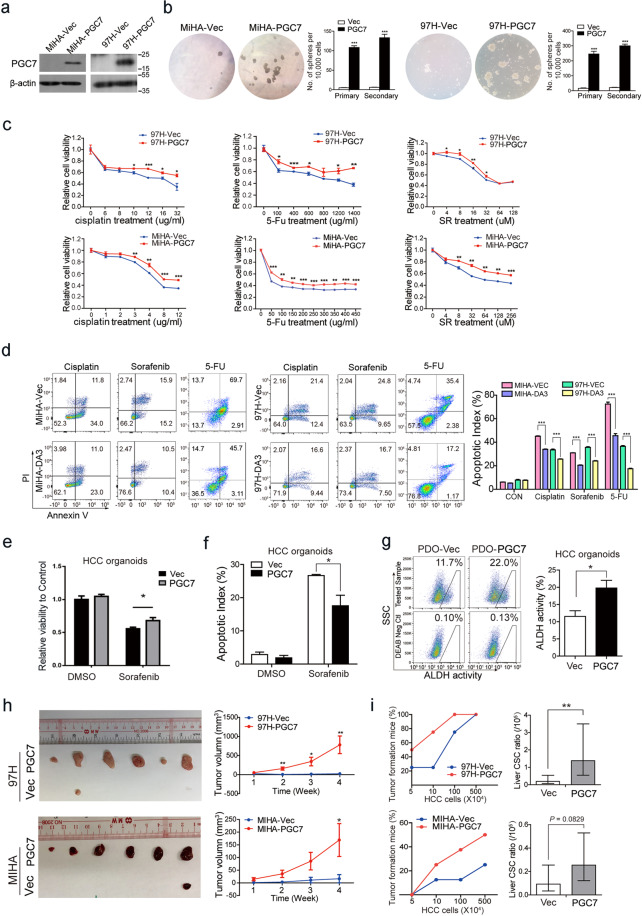
PGC7 regulates HCC oncogenic dedifferentiation both in vitro and in vivo. **a** Overexpression of PGC7 was detected by western blotting in PGC7- and empty vector (Vec)-transfected MIHA and 97H cells. β-actin was used as a loading control. Three independent experiments were conducted. **b** PGC7 enhanced sphere formation activity in both MIHA and 97H cells. Calculated ratios were shown in the right panel. **c** Cell viability between Vec- and PGC7-transfected cells was compared by XTT assay after treatment with cisplatin, 5-Fu, and sorafenib at the indicated concentration for 48 h. **d** Apoptotic indexes between Vec- and PGC7-transfected cells were compared by flow cytometry with Annexin-V-fluorescein isothiocyanate double staining after treatment with cisplatin, sorafenib, or 5-Fu at the indicated concentration for 24 h. The apoptotic index was defined as the percentage of apoptotic cells. **e** Cell viability between Vec- and PGC7-transfected HCC organoids were compared by CellTiter-Glo assay after treatment with 8 μM sorafenib for 5 days. **f** Apoptotic index between Vec- and PGC7-transfected HCC organoids were compared by flow cytometry after treatment with 8 μM sorafenib for 5 days. **g** Flow cytometry analysis for ALDH activity using the ALDEFLUOR kit in Vec- and PGC7-transfected HCC organoids. DEAB stands for negative control when cells were treated with an ALDH inhibitor, diethylaminobenzaldehyde. **h** Vec- and PGC7-transfected MIHA (5 × 10^6^, 1:1 matrigel mixture) and 97H cells (5 × 10^6^) were injected into BALB/c nude mice. Tumor sizes were observed every week. The average tumor volume was expressed as the mean ± SD of 6 mice. **i** PGC7 overexpression enhanced tumor-initiating capacity. Vec- and PGC7-transfected 97H and MIHA cells with different dilution ratios were implanted into NOD/SCID mice for tumor initiation. Percentages of tumor-formation mice were calculated (left panel), and the frequency of stem cells (liver CSC ratio) were analyzed using ELDA software (right panel). Error bars represent the upper/lower limit. Statistics: in **b**–**h**, Student’s *t*-test was used for statistical analysis, *P < 0.05, **P < 0.01, ***P < 0.001, data are shown as mean ± SD. Data represent at least three independent experiment.

To investigate the role of PGC7 in HCC tumor differentiation and lineage reversion, we established several cell lines with PGC7 overexpressing or silencing. Western blot and immunofluorescence staining confirmed the exogenous expression of PGC7 and its nuclear localization (Fig. 2a and Supplementary Fig. 2a). Lineage progenitor cells usually exhibited enhanced self-renewal ability. We found that overexpression of PGC7 markedly improved both primary and secondary sphere formation ability in MIHA and 97H cell lines (Fig. 2b). Conversely, knockdown of PGC7 significantly decreased the size and number of spheres formed in PLC-8024 cells (Supplementary Fig. 2b, c). Pluripotent markers were found upregulated in 3D spheroids (Supplementary Fig. 2d), among which NANOG, SOX2, and OCT4 exhibited increased expression in PGC7-transfected cells (Supplementary Fig. 2e). Low sensitivity to chemotherapeutic drugs is one of the most important hallmarks of tumor progenitor cells and maintains the major challenge in HCC treatment. To test whether PGC7 affects the sensitivity of HCC cells to chemotherapeutic or targeted drugs, PGC7-transfected cells were treated with cisplatin, 5-fluorouracil (5-Fu), and sorafenib. Cell viability and flow cytometry analysis showed that the frequencies of apoptotic cells were significantly decreased in PGC7-transfected MIHA and 97H cells (Fig. 2c, d), as well as HCC patient-derived organoids (PDOs) (Fig. 2e, f), indicating PGC7 confers chemoresistance property to HCC cells. Conversely, silencing of PGC7 markedly inhibited viability and increased the proportions of apoptotic cells (Supplementary Fig. 2f, g). The effect on drug-sensitivity was also confirmed in vivo using the xenograft mice model. Tumors induced by PGC7-overexpressed cells showed retarded response to sorafenib treatment, compared with Vec-transfected cells (Supplementary Fig. 2h). As high ALDH (aldehyde dehydrogenase) activity serves as a universal marker of lineage precursor cells [21], we then tested ALDH activity in PDOs transfected with PGC7. Compared with Vec-transfected PDOs, PGC7-transfected PDOs demonstrated enhanced ALDH activity (Fig. 2g).

To further confirm whether PGC7 could promote HCC lineage reversion in vivo, empty vector- and PGC7-transfected cells were subcutaneously injected into the left and right dorsal flank of nude mice (*n* = 6). Results showed the PGC7-transfected cells overtly increased xenograft tumor growth (Fig. 2h). In contrast, no tumor formation was observed for PLC-8024 cells with PGC7 silencing (Supplementary Fig. 2i). We next subcutaneously implanted 500×, 100×, 10×, and 5 × 10^4^ cells into NOD/SCID mice. PGC7-transfected cells demonstrated enhanced tumor-initiating capacity and increased tumor progenitor ratio (Fig. 2i). Moreover, IHC staining was performed in the xenograft tumors and stronger intensity of AFP and CK19 were found in tumors induced by PGC7-overexpressed cells compared with Vec-transfected cells (Supplementary Fig. 2j). To summarize, these data suggest that PGC7 is involved in regulating HCC lineage reversion both in vitro and in vivo.

### PGC7 remodels DNA methylation patterns for key developmental transcription factors related to pluripotency and differentiation

Because PGC7 has been reported to preserve DNA methylation pattern in either hyper- or hypomethylated status of early embryos or mice oocyte [9, 11], we propose that PGC7 may exert its function on HCC cells through regulating DNA methylation. Whole-genome bisulfite genomic sequencing (WGBS) was performed to compare the methylation status between Vec- and PGC7-transfected MIHA cells. The total sequence yield was 89.67 and 96.11 gigabases (Gb), with an average read depth of 21.63 and 23.42 per strand (Supplementary Fig. 3a). In each cell type, over 94% of the cytosines in the genome were covered by at least one read depth (Supplementary Fig. 3b). Approximately 24 million and 28 million methylcytosines were detected in Vec- and PGC7-transfected cells respectively, and over 96% of the methylcytosines were in the CG context (Supplementary Fig. 3c).

**Fig. 3:**
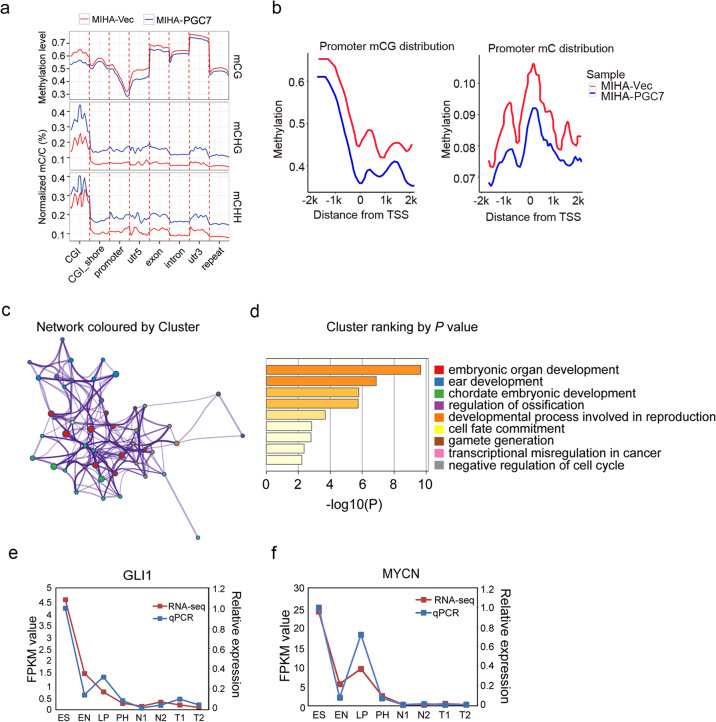
PGC7 facilitates promoter demethylation of key transcriptional factors associated with development and differentiation. **a** Methylation level or relative methylation density (the average density of methylation relative to the underlying density of all potential sites of methylation) in Vec- and PGC7-transfected MIHA cells throughout different gene-associated regions in each cytosine context (mCG, mCHG, mCHH). The mean mC/C profile was normalized to the maximum value. **b** Average methylation level of cytosines (mCG and mC) across promoter regions were compared between Vec- and PGC7-transfected cells (promoters encompass ±2 kb from the transcription starting site). **c**, **d** Enrichment analysis using Metascape (https://metascape.org/gp/index.html#/main/step1) revealed that the activated transcription factors formed an interactive network (**c**) characterized by gene clusters associated with the developmental process (**d**). Significantly enriched clusters marked in different colors in the network were annotated in figure (**d**). **e**, **f** qRT-PCR was used to verify the gene expression pattern of GLI1 and MYCN in the model of in vitro hepatocyte differentiation. Data were shown as the mean of three independent experiments.

To examine the difference of methylation distribution between Vec- and PGC7-transfected cells, we firstly compared the methylation level and density throughout various genomic features. The results revealed PGC7 introduction decreased the methylation level of mCG, while increased the relative methylation density of non-CG (mCHG and mCHH, where H = A, C, or T) across all the genomic features (Fig. 3a). The result is consistent with the theory that non-CG methylation primarily exists in pluripotent stem cells and disappeared after differentiation [22]. By looking at the promoter regions (±2 kb to the TSS), lower methylation densities of mCG and mC were observed in PGC7-overexpressed cells, indicating the transcription activity was elevated (Fig. 3b). To identify differentially methylated promoters (DMPs), a sliding window approach was used to scan the genome and 537 genes with promoter demethylation were discovered (Supplementary Table 2). Bioinformatics-aided analysis using Metascape [23] revealed that the potential targets of PGC7 formed clusters associated with cell cycle, CDC42 signaling, and PLK1 pathway (Supplementary Fig. 3d, e). Interestingly, by analyzing the 537 genes with promoter demethylation, we found 44 of them were transcription factors and formed a regulatory network of embryonic development, cell fate commitment, and transcriptional misregulation in cancer (Fig. 3c, d). This evidence implied that PGC7 might activate the oncogenic developmental network through remodeling DNA methylation patterns in HCC.

### PGC7 activates key transcription factors to regulate the self-renewal and differentiation of HCC

Expression of transcription factors showing promoter demethylation in PGC7-overexpressed cells was analyzed by qRT-PCR. Among the potential PGC7 targets, GLI1 and MYCN are the most significantly upregulated transcription factors (Supplementary Table 3). GLI1 belongs to the GLI family proteins and has been shown to be involved in cell fate determination [24]. The MYCN gene is a member of the MYC family and was reported to be important in regulating the developmental process and oncogenesis [25]. In our hepatocyte differentiation model, MYCN shared a similar expression pattern with PGC7, and GLI1 was highly expressed in embryonic stem cells and decreased with differentiation, indicating their functions in pluripotency maintenance lineage reversion (Fig. 3e, f).

**Fig. 4:**
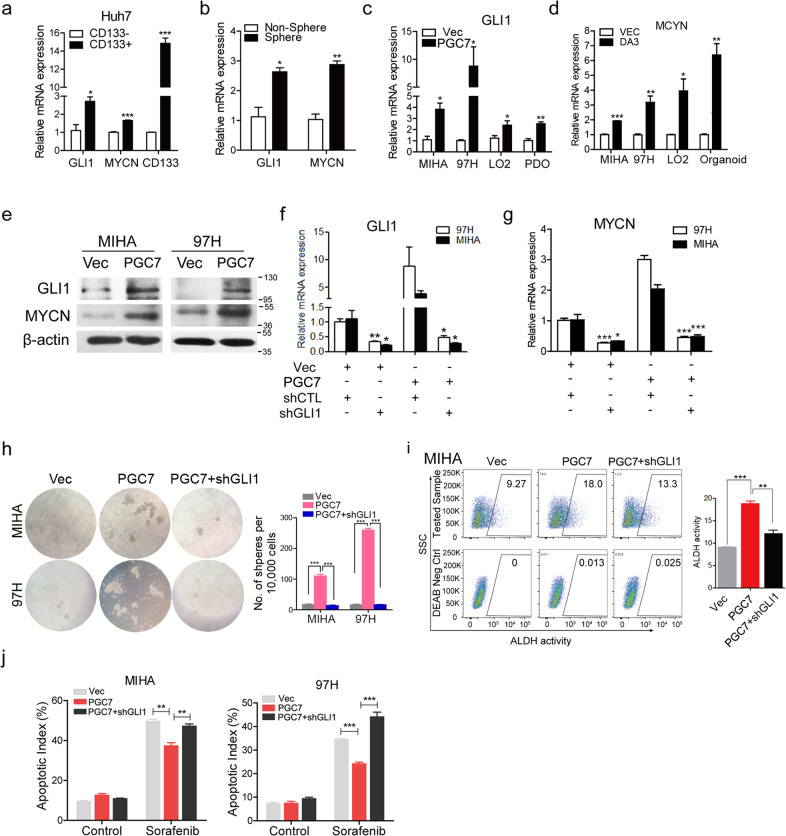
PGC7 activates key developmental transcription factors to regulate self-renewal and differentiation. **a**, **b** GLI1 and MYNC expression were upregulated in CD133^+^ populations sorted from Huh7 cells (**a**), and in spheroids formed from 97H cells (**b**) measured by qRT-PCR. **c**, **d** PGC7 increased the expression of GLI1 (**c**) and MYCN (**d**) in cell lines MIHA, 97H, LO2 as well as HCC organoids. PDO patient-derived organoids. **e** Upregulation of GLI1 and MYCN by PGC7 was confirmed by western blotting in MIHA and 97H cells. β-actin was used as a loading control. Three independent experiments were conducted. **f**, **g** shRNA was applied to silence GLI1 expression (shGLI1) in both Vec- and PGC7-transfected cells. shCTL was the empty vector without insertion of any shRNA sequence. The expression of GLI1 (**f**) and MYCN (**g**) was detected by qRT-PCR. **h**–**j** GLI1-silenced 97H and MIHA cells in the absence or presence of PGC7 were cultured for sphere formation assays (**h**), ALDH activity characterization (**i**), as well as apoptotic assay with sorafenib treatment for 24 h (**j**). DEAB stands for negative control when cells were treated with an ALDH inhibitor diethylaminobenzaldehyde. The negative control was used to help gating the ALDH positive cells in the experimental group. Statistics: in **a**–**d** and **f**–**j**, Student’s *t*-test was used for statistical analysis, *P < 0.05, **P < 0.01, ***P < 0.001, data are shown as mean ± SD. Data represent at least three independent experiment.

**Fig. 5:**
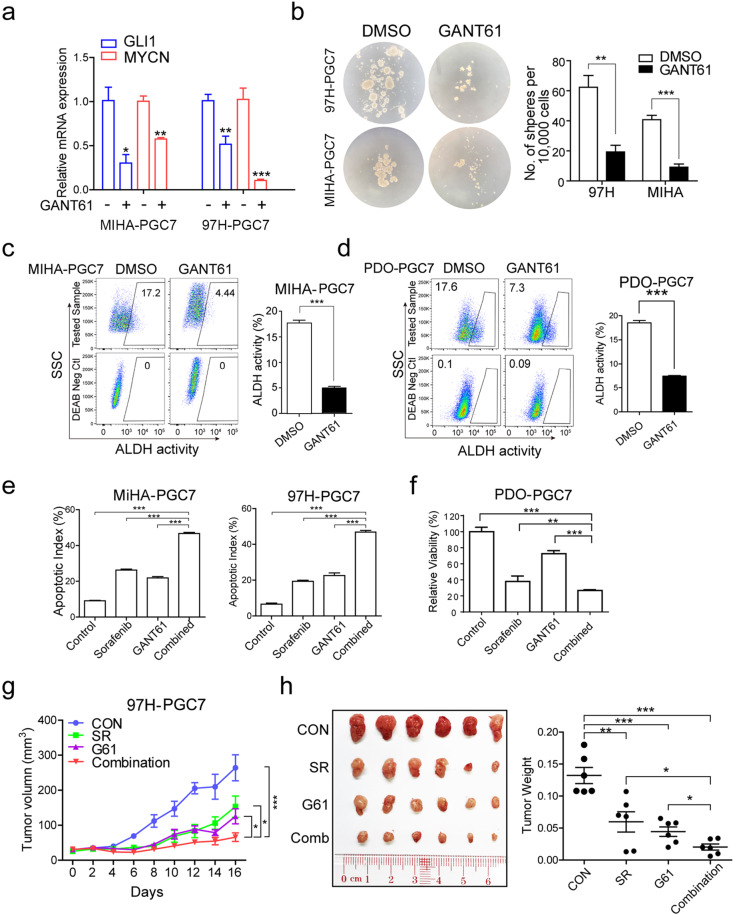
GLI1 inhibitor GANT61 impaired the function of PGC7 on tumor lineage reversion both in vitro and in vivo. **a** Expression of GLI1 and MYCN was detected by qRT-PCR with the treatment of 10 μM GANT61 for 48 h. **b**–**d** PGC7 overexpressing cells were pretreated with 10 μM GANT61 or DMSO solvent for 2 days, followed by sphere formation (**b**), ALDH activity characterization in MIHA cells (**c**), and in HCC organoids (**d**). **e** PGC7-overexpressed 97H and MIHA was treated with vehicle control, sorafenib (16 μM), GANT61 (10 μM), or the combined treatment of both drugs for 2 days, followed by apoptotic index detection. **f** PGC7-overexpressed HCC organoids were treated with vehicle control, sorafenib (8 μM), GANT61 (10 μM), or the combined treatment of both drugs for 5 days, and cell viability was detected by CellTiter-Glo assay. **g** Mice with established subcutaneous HCC tumors of similar size were randomly divided into four groups and were given vehicle control, 10 mg/kg sorafenib via oral gavage, 50 mg/kg GANT61 via intraperitoneal injection, or combined treatment. Sorafenib was given daily and GANT61 was administered every other day. The average tumor volume was expressed as the mean ± SD of 6 mice. **h** The tumors at the end of treatment (left panel) and a graph showing the weight of tumors at the end of treatment (right panel). Each dot represents a single tumor. Statistics: in **a**–**h**, Student’s *t*-test was used for statistical analysis, *P < 0.05, **P < 0.01, ***P < 0.001, data are shown as mean ± SD. Data represent at least three independent experiment.

To explore the role of GLI1/MYCN in HCC, we examined their expression levels in CD133^+^ CSCs and oncospheres derived from Huh7 cells. Both of them were significantly enriched in liver CSCs (Fig. 4a, b). Their upregulation induced by PGC7 was also confirmed in multiple cell lines and HCC organoids in both mRNA (Fig. 4c, d) and protein level (Fig. 4e), while decreased expression was observed in PGC7-silenced HCC cells (Supplementary Fig. 4a). Notably, MYCN was discovered as a downstream target of GLI1 according to the TRANSFAC database based on CHIP-seq analysis and literature mining. To determine whether PGC7 promotes lineage reversion in a GLI1-dependent manner, shRNA was used to knockdown GLI1 expression in HCC cells. Silencing GLI1 in both Vec- and PGC7-transfected 97H and MIHA cells or in parental PLC-8024 cells remarkably suppressed MYCN expression (Fig. 4f, g and Supplementary Fig. 4b), verifying MYCN as a downstream target gene of GLI1 in HCC. Functional assays including sphere formation (Fig. 4h), ALDH activity (Fig. 4i), and sorafenib-induced apoptosis (Fig. 4j) were then applied and results showed that GLI1 knockdown significantly impaired the progenitor-like features of HCC cells induced by PGC7. We next tested whether the inhibitor of GLI1 (GANT61) exerts a similar effect as GLI1 knockdown. After treatment of 5 μM GANT61 for 3 days, HCC cells demonstrated a reduction of GLI1 and MYCN expression (Fig. 5a and Supplementary Fig. 5a). Fewer spheres (Fig. 5b) and lower ALDH activity (Fig. 5c, d) were observed after GANT61 treatment in HCC cell lines and organoids with PGC7 ectopic expression. To investigate the effect of MYCN on HCC self-renewal, the inhibitor JQ1 was applied in PLC-8024 cells. Treatment of 6 μM JQ1 for 3 days evidently reduced the expression of MYCN but not GLI1 (Supplementary Fig. 5b) and suppressed the sphere formation activity (Supplementary Fig. 5c). These results indicate that PGC7 promotes lineage reversion of HCC via GLI1/MYCN activation.

### Combined treatment of sorafenib with GANT61 results in maximal tumor suppression both in vitro and in vivo

To examine the role of GLI1 in regulating sorafenib resistance induced by PGC7, we treated PGC7-overexpressed HCC cells and organoids with both sorafenib and GANT61. Results showed GLI1 inhibition sensitized HCC cells (Fig. 5e) and organoids (Fig. 5f) to sorafenib treatment. Given the crucial role of GLI1 in regulating sorafenib resistance, we evaluated the therapeutic effect of GANT61 treatment and its combined effect with sorafenib in vivo using PGC7-transfected HCC cells. Mice with established tumors were given either vehicle control, GANT61, sorafenib, or the combination of both for 16 days. Compared with the vehicle control group, GANT61, sorafenib, and combined treatment groups all demonstrated reduced tumor size and weight. Besides, the combined treatment group showed maximal tumor suppression (Fig. 5g, h), while no significant difference in mice body weight was observed (Supplementary Fig. 5d). The therapeutic potential of JQ1 was also investigated. Treatment of 10 μM JQ1 for 2 days sensitized HCC cells to sorafenib-induced apoptosis (Supplementary Fig. 5e). Combined treatment of JQ1 with sorafenib in xenograft mice achieved the maximum tumor suppression (Supplementary Fig. 5f, g). These findings indicated that inhibition of PGC7/GLI1/MYCN might reverse poorly differentiated HCCs and provide new therapeutic strategies.

### PGC7 interacts with UHRF1 to induce promoter demethylation of key transcription factor

**Fig. 6:**
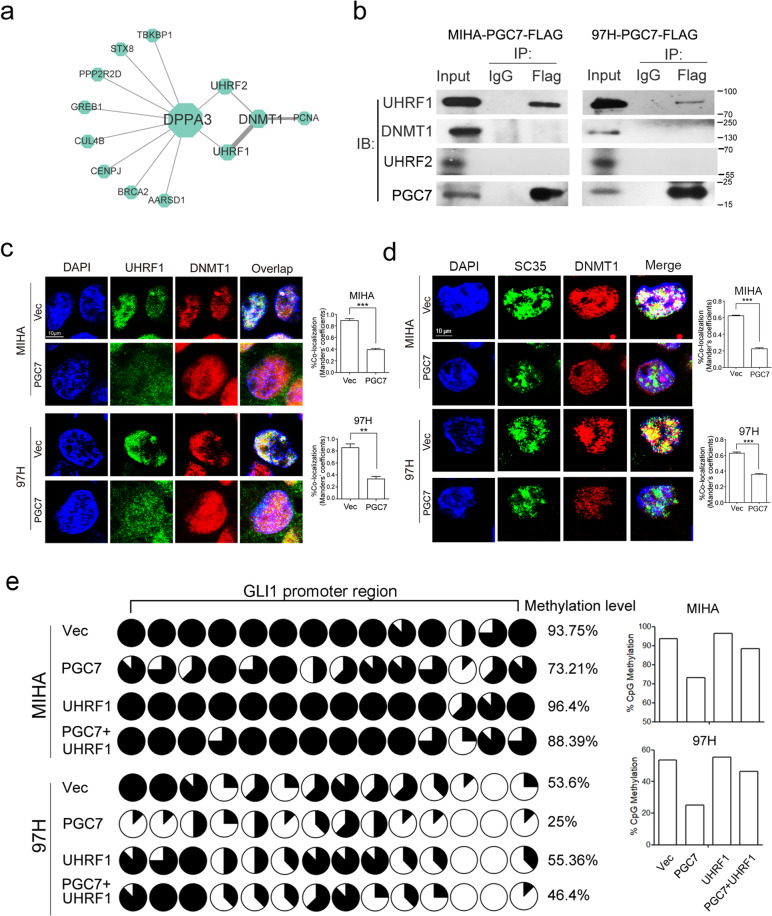
PGC7 interacts with UHRF1 to induce GLI1 promoter demethylation. **a** Protein interactions of PGC7 illustrated by Cytoscape. **b** Flag-tagged PGC7 was expressed in MIHA and 97H cells. PGC7 and UHRF1 were immunoprecipitated with anti-Flag antibodies. The immunoprecipitates were analyzed by western blotting. Three independent experiments were conducted. **c** Immunofluorescence analysis of UHRF1 and DNMT1 in Vec- and PGC7-transfected MIHA and 97H cells. UHRF1 and DNMT1 were shown in green and red, respectively; nuclei were counterstained with DAPI (blue) (left panel). Bar charts (right panel) showed the percentage of colocalization (Manders’ coefficients) of UHRF1 and DNMT1 in MIHA and 97H cells. Results were obtained from three independent experiments. Student’s *t*-test was used for statistical analysis, **P < 0.01, ***P < 0.001. **d** Immunofluorescent staining of DNMT1 (red) and SC35 (green) in Vec- and PGC7-transfected MIHA and 97H cells (left panel). Bar charts (right panel) showed the percentage of colocalization (Manders’ coefficients) of DNMT1 and SC35 staining. Student’s *t*-test was used for statistical analysis, ***P < 0.001. **e** The promoter region of GLI1 was analyzed and the methylation status of CpG dinucleotides in cells transfected with empty vector, PGC7, UHRF1, and co-transfected with PGC7 and UHRF1 was detected by bisulfite genomic sequencing (BGS). The percentage of methylation at each CpG site was displayed in the pie charts (left panel). The average methylation level in the promoter region was calculated and shown in the bar chart (right panel).

To decipher the mechanism of PGC7 in regulating DNA methylation, we analyzed the interacting proteins of PGC7 using the BioGrid platform (https://thebiogrid.org/). Cytoscape was then used to depict the protein interactions according to evidence value (Fig. 6a). Among the proteins that interacted with PGC7, UHRF1 and UHRF2 arouse our interest with their association with DNA methylation. Immunoprecipitation analysis demonstrated PGC7 binds to UHRF1 rather than UHRF2 or DNMT1 in PGC7-overexpressed 97H and MIHA cells (Fig. 6b and Supplementary Fig. 6a). UHRF1 has been reported to maintain DNA methylation via DNMT1 recruitment [26]. To assess the molecular mechanism more specifically, we investigated the nuclear localization of UHRF1 and DNMT1 in cells synchronized to the S phase (Supplementary Fig. 6b) by dual immunofluorescence staining. We found ectopic expression of PGC7 sequestered UHRF1 from the nuclei to the cytoplasm (Fig. 6c and Supplementary Fig. 6c), while the loss of PGC7-induced UHRF1 nuclear enrichment (Supplementary Fig. 6d). The quantification result exhibited that the percentage of UHRF1 and DNMT1 colocalization in control cells (over 80%) was significantly higher than that in PGC7 expressing cells (<40%) in both cell lines (Fig. 6c, right panel). The nuclear sub-localization of DNMT1 was also investigated and the results showed PGC7 disturbed the localization of DNMT1 on the nuclear speckles (SC35 staining) in both cell lines (Fig. 6d), indicating UHRF1–DNMT1 function was disturbed by PGC7 in HCC cells.

Then, we studied the effect of UHRF1 on PGC7-induced promoter demethylation. Bisulfite genomic sequencing in the promoter region of GLI1 was conducted. The result showed that overexpression of UHRF1 could abolish the effect of PGC7-induced GLI1 promoter demethylation but had no effect on Vec-transfected HCC cells (Fig. 6e and Supplementary Fig. 6e). Conversely, silencing of PGC7 increased the methylation level of the GLI1 promoter (Supplementary Fig. 6f, g). To investigate the role of UHRF1 in regulating PGC7-mediated HCC lineage reversion, sphere formation, and xenograft mice experiments were performed. Fewer spheroids and smaller tumors were generated by 97H-PGC7 cells with UHRF1 ectopic expression, compared with cells without UHRF1 expression (Supplementary Fig. 6h, i). Collectively, these data strongly suggested that binding of PGC7 to UHRF1 disrupts the localization of maintenance DNA methyltransferase DNMT1 in the nucleus, thus results in GLI1 promoter demethylation and HCC lineage reversion (Supplementary Fig. 6j).

### GLI1/MYCN activation is correlated with clinical severity and prognosis of HCC patients

**Fig. 7:**
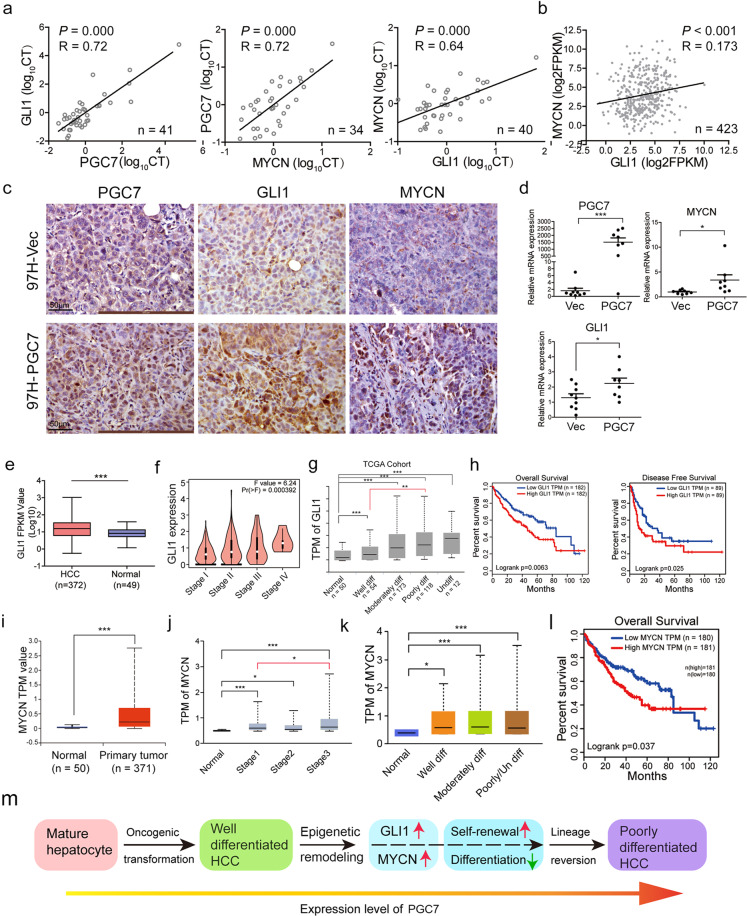
Clinical relevance of PGC7/GLI1/MYCN in HCC oncogenic dedifferentiation. **a** The association among PGC7, GLI1, and MYCN in HCC clinical specimens was detected by qRT-PCR. Pearson coefficient R was used to denote the expression correlation between any of the two genes. **b** The expression association of GLI1 and MYCN in the TCGA database was analyzed. Pearson coefficient *R* was used to denote the expression correlation. **c** Representative images of IHC staining of PGC7, GLI1, and MYCN in xenograft mice tumor generated by Vec- and PGC7-transfected 97H cells. Three independent experiments were conducted. **d** The mRNA expression of PGC7, GLI1, and MYCN were detected in xenograft tumor tissues generated by Vec- and PGC7-transfected cells. Each dot represents a single tumor. **e**–**g** The expression of GLI1 was analyzed in TCGA cohorts divided by tumor and non-tumor (**e**), different clinicopathological stage (**f**), and differentiation status (**g**). **h** Kaplan–Meier survival analysis in TCGA cohorts showed HCC patients with GLI1 higher expression (*n* = 182) had worse overall survival and disease-free survival outcome compared with patients having lower GLI1 expression (*n* = 182). **i**–**k** The expression of MYCN was analyzed in TCGA cohorts divided by tumor and non-tumor (**i**), different clinicopathological stage (**j**), and differentiation status (**k**). **l** Overall survival analysis of HCC patients with high or low MYCN expression in TCGA cohorts. **m** A schematic diagram showing the proposed working model of PGC7 in HCC lineage reversion, illustrating the dynamic expression of PGC7 during liver development and HCC progression. Statistics: in **d**–**g** and **i**, Student’s *t*-test was used for statistical analysis, *P < 0.05, **P < 0.01, ***P < 0.001, data are shown as mean ± SD.

As GLI1 and MYCN are two important downstream targets of PGC7 in HCC cells, we proceeded to investigate their co-expression pattern in HCC. An association study showed that PGC7 expression was positively correlated with GLI1 (Pearson *R* = 0.72; *P* = 0.000) and MYCN (Pearson *R* = 0.72; *P* = 0.000) in our own HCC cohort (Fig. 7a). As expected, the expression of GLI1 was also positively correlated with MYCN both in our HCC cohort (Pearson *R* = 0.64; *P* = 0.000, Fig. 7a) and TCGA database (Pearson *R* = 0.173; *P* < 0.001, Fig. 7b). GLI1 and MYCN expression was further examined in xenograft tumors. IHC staining (Fig. 7c) and qRT-PCR (Fig. 7d) results demonstrated that both GLI1 and MYCN were markedly increased in xenograft tumors induced by PGC7-transfected HCC cells. Double immunofluorescence staining revealed the colocalization pattern of GLI1 and MYCN in xenograft tumors with PGC7 overexpression (tM1 = 0.52, Supplementary Fig. 7).

Since GLI1/MYCN had a crucial role in HCC lineage reversion, the relationship of GLI1/MYCN activation with HCC progression was further explored. We observed GLI1 was highly expressed in the tumor tissues of HCC patients in the TCGA database (Fig. 7e), and the expression levels were consistent with clinicopathological stages (Fig. 7f). Importantly, GLI1 expression increased progressively from well-differentiated tumors to poor or undifferentiated ones (Fig. 7g) and was associated with poor prognosis (Fig. 7h). Similar observations were achieved when analyzing MYCN expression in the TCGA database. The expression of MYCN was increased gradually with tumor stage development and upregulated in poor or undifferentiated tumors compared with normal tissues (Fig. 7i–k), which was significantly associated with poor clinical outcome (Fig. 7l). In summary, we found that PGC7 is dynamically expressed during liver development and HCC progression. PGC7 was highly expressed in the fetal liver, decreased during hepatocyte maturation, then reactivated in HCCs, and increased progressively from well-differentiated tumors to poorly differentiated tumors. Ectopic expression of PGC7 in HCC induces promoter demethylation of key developmental transcriptional factors and activates their transcription. This further enables HCC cells to acquire lineage progenitor-like features including enhanced self-renewal and drug resistance, which have significantly contributed to the aggressiveness of HCC (Fig. 7m).

## Discussion

Accumulating evidence indicated that cancer malignant transformation is associated with the activation of tumor precursor cells [27]. In the process of organ development, lineage-specific regulators remain active and gradually decrease until the terminal differentiation. However, in some cases they were found reactivated in tumor cells, conferring precursor-like features and contributing to cancer progression [28]. Epithelial-to-mesenchymal transition (EMT) is one of the evidence indicating the association between developmental signaling and cancer progression [29, 30]. To investigate the reactivation of key developmental regulators in HCC, we established an in vitro hepatocyte differentiation model, in which hESCs were differentiated into human hepatocytes via several stages. With this model, we could easily identify genes most active in the liver progenitor stage, gradually silenced in mature hepatocytes and reactivated in HCC, indicating their roles in regulating lineage reversion of HCC.

PGC7, also known as DPPA3 and STELLA, was initially identified to be preferentially expressed in primordial germ cells (PGCs) [31]. The chromosomal location of PGC7 is 12p13.31, a region consisting of conserved gene clusters including NANOG and GDF3 [7], which are highly expressed in fetal germ cells and hESCs [32]. Notably, PGC7 expression emerged earlier than endogenous pluripotency genes including Oct3/4, Sox2, Nanog, and Klf4 during somatic cell reprogramming, and is indispensable for the generation of fully reprogrammed iPSCs [13, 33]. All the evidence suggests PGC7 has a critical role in pluripotency maintenance. Here we demonstrate that PGC7 expression reached its peak value in the liver progenitor stage, then gradually disappeared in mature hepatocytes and reactivated in HCC tumor. Our histological and functional studies reveal that PGC7 expression is enriched in liver CSCs and conferred progenitor-like features in HCC.

Based on previous literature, the mechanism of how PGC7 regulates DNA methylation status remains controversial. Some studies conclude that PGC7 protects DNA methylation of imprinted loci and is essential for early embryo development [9, 10]. A recent study demonstrated PGC7 maintains the mice oocyte genome at a hypomethylated status via preventing de novo DNA methylation mediated by DNMT1 and UHRF1 [11]. Here we demonstrated a single-base-resolution map of DNA methylation in liver cells after ectopic expression of PGC7. We found that the average mCG level was decreased by PGC7 across various genomic features including promoter regions. Mechanistically, we observed PGC7 overexpression sequestered UHRF1 from the nucleus, disturbing the correct localization and function of DNMT1. However, several challenges still remain regarding the detailed interactive mode between PGC7 and UHRF1. For example, how PGC7 sequestered UHRF1 from the nucleus when it was predominantly expressed in the nucleus? One possible explanation is PGC7 shuttles between nuclei and cytosol. Upon overexpression, the amount of PGC7 protein is far more abundant than UHRF1, leading to UHRF1 export regardless of the pattern of PGC7 distribution. In this situation, the uncoupling mechanism in the cytosol that releases UHRF1 from PGC7 is yet to be determined.

GLI family proteins are the ultimate effectors of Hedgehog signaling. As zinc-finger transcription factors, GLI1 was reported to induce Hh target genes, having essential roles in embryonic development, tumorigenesis, and maintenance of CSCs [34, 35]. In the present study, we found MYCN is one of the downstream targets of GLI1, and both of them could be activated via promoter demethylation induced by PGC7. Small-molecule inhibitor GANT61 [36] was used to block GLI-mediated transcription, and the self-renewal features of HCC cells were suppressed. It is important to notice that the application of GANT61 increased the sensitivity of Sorafenib both in vitro and in vivo, suggesting that GANT61 might be used as a novel inhibitor to augment the efficacy of sorafenib in HCC treatment.

Above all, evidence from in vitro, in vivo, patient-derived organoids and clinical data analysis suggested an important role of PGC7 in HCC lineage reversion and malignant progression. Further inhibition of PGC7 and the related developmental signaling networks might help to reverse poorly differentiated HCCs and sensitize them to chemotherapeutic or targeted drugs. This might provide a novel therapeutic strategy in HCC treatment.

## Supplementary information

Supplementary materials and methods

Supplementary Figure 1

Supplementary Figure 2

Supplementary Figure 3

Supplementary Figure 4

Supplementary Figure 5

Supplementary Figure 6

Supplementary Figure 7

Supplementary table 1

Supplementary table 2

Supplementary table 3

## Data Availability

The data sets used and/or analyzed during the current study are available from the corresponding author on reasonable request.
