# Supplementary material for: PGC7 promotes tumor oncogenic dedifferentiation through remodeling DNA methylation pattern for key developmental transcription factors
Source: Cell Death Differ. 2021 Jan 26;28(6):1955–70. doi: 10.1038/s41418-020-00726-3 (PMC8185079; doi:10.1038/s41418-020-00726-3)
Supplement: Supplementary file 1 — Supplementary materials and methods [file 41418_2020_726_MOESM1_ESM.docx]

**Supplementary materials and methods**

**Antibodies and Reagents**

Commercial antibodies were mouse anti-Flag, mouse anti-ß-actin (Sigma-Aldrich); mouse anti-PGC7, mouse anti-UHRF1 (Santa Cruz Biotechnology); Rabbit anti-DNMT1, Rabbit anti-GLI1, Rabbit anti-MYCN (Abcam); PE-conjugated CD133 antibody (MACS Miltenyi Biotec); Alexa488-conjugated goat anti-mouse immunoglobulin-G (IgG), Alexa555-conjugated goat anti-rabbit IgG (Invitrogen); Dilutions of anti-ß-actin, anti-PGC7, anti-GLI1, anti-MYCN for western blot were 1:1000, for IHC were 1:200; Dilutions of fluorescence-conjugated secondary antibodies for immunofluorescence staining were 1:300. Other major reagents were bFGF (Millipore), EGF (Sigma-Aldrich), insulin (Life Technologies), B27 (Life Technologies), DAPI (Sigma-Aldrich), and GNAT61 (Sigma-Aldrich).

**Lentivirus production and cell infection**

We constructed PCDH-Puro, PCDH-GFP Vector and PCDH-Puro-PGC7, PCDH-GFP-PGC7 plasmid, and transfected 293FT cells for virus production. HCC cells or immortalized liver cells were infected by the virus supernatants. After drug screening or GFP sorting, we established PGC7 overexpression cells. GLI1-silenced HCC cells were established using the PLL3.7 shRNA vectors by similar strategy. The sequence of shRNA targeting GLI1 was: 5’–GCTCAGCTTGTGTGTAATTAT– 3’.

**RNA extraction and quantitative RT-PCR**

Total RNA was extracted using TRIZOL reagent (Invitrogen). Briefly, fresh specimens were homogenized with Trizol, and then segregated by adding chloroform. After obtaining the aqueous phase, isopropanol and 75% ethanol were added sequentially. RNA pellet was dissolved in DEPC water. Transcriptor High Fidelity cDNA synthesis Kit (Roche) was used to prepare cDNA from RNA. SYBR Green PCR Kit (Applied Biosystems) and ABI 7900HT Sequence Detector (Applied Biosystems) were applied to conduct qRT-PCR. Specificity of primers was verified by dissociation curve analysis. Data were analyzed using ABI SDS software. Primer sequences are listed in Supplementary Table 1.

**Western blot**

For western blot, cells were homogenized with RIPA buffer (Thermo Scientific). Together with protein loading dye (Bio-Rad), they were boiled for 15 min. Supernatants were loaded onto SDS-PAGE gels. Nitrocellulose membranes were incubated with primary antibodies at 4°C for overnight and then incubated and visualized by HRP conjugated secondary antibodies.

**IHC staining**

IHC staining was performed using the standard streptavidin-biotin-peroxidase complex method. Briefly, paraffin sections were deparaffinized and rehydrated. Tissues were boiled for antigen retrieval for 45 min in appropriate buffer (Dako). Then sections were incubated with primary antibodies at 4°C for overnight. After washing three times with TBS, sections were incubated in HRP-conjugated secondary antibodies (1:300 dilution), and the subsequent detection was performed using the standard substrate detection of HRP (DAB, DAKO), followed by hematoxylin counterstaining. Stained tissues were image on an AperioScanscope CS imager (Vista, CA, USA). Investigators were blinded for

**Immunoprecipitation Assays**

For immunoprecipitation assay, 5 mg of total cell lysate was immunoprecipitated with anti-FLAG or anti-IgG Affinity gel Sigma-Aldrich) at 4°C for overnight. Extensive washing and immunocomplexes denaturation steps were carried out according to the manufacture’s instruction (ROCHE). Denatured immunocomplexes were analyzed by western blotting. About 5% of the whole lysate (Input) was used as a positive control. Western blotting analysis was performed with the standard protocol.

**Flow cytometry and cell sorting**

For nuclear protein staining by flow cytometry, cells were digested with trypsin and resuspended in 100 µl PBS. Add 500 µl permeabilization buffer (0.5% Triton X-100, 0.2 µg/ml EDTA and 1% BSA in PBS) and incubate for 15 minutes on ice. Add 3 ml of ice-cold 100% methanol and incubate for 10 minutes, and samples were proceeded with staining. After being labeled with primary and secondary antibodies, samples were washed three times and analyzed with BD LSR Fortessa Analyzer (BD Biosciences). For CD133 cell sorting, cells were suspended in PBS containing 2% FBS with PE-conjugated anti-CD133 antibody. Isotype-matched rabbit IgG served as controls to gate positive cells. Samples were sorted on FACSAria I Cell Sorter (BD Biosciences), and data analyzed using FlowJo software. Only the top or bottom 10% cells with strongest or weakest staining were gated as CD133 positive and negative populations, respectively.

**Immunofluorescence**

Immunofluorescence was performed on cell lines and cryosectioned spheroids. First, cells on the coverslips were fixed with 4% paraformaldehyde, and incubated with primary antibody at 4°C for overnight. After washing, cells underwent incubation with AlexaFluor dye-conjugated secondary antibodies, followed by counterstaining with DAPI for 10 min at room temperature. Images were acquired using laser scanning confocal microscope.

**Sphere formation assay**

3,000 of HCC cells were seed in Ultra Low Attachment 24-well plates (Corning Incorporated Life Sciences, MA, USA) and cultured in serum-free DMEM/F12 medium supplemented with B27 (1:50), 4 μg/ml insulin, 20 ng/ml human recombinant EGF, 10 ng/ml human recombinant basic FGF. Cells were replenished with fresh medium every second day. For serial passage of primary spheroids, the primary spheroids were collected and dissociated into single cells using TrypLE (Invitrogen). Following dissociation, trypsin inhibitor (Invitrogen) was used to neutralize the reaction and cells were resuspended in the same medium with supplements. For non-sphere cell separation, we collected all cells in Falcon tube and let stand for 5 minutes. Pellets were spheres and we transfer the supernatants into a new falcon tube followed by centrifugation at 1,500g for 5 min. Pellets here were non-spheres and used directly for subsequent experiments.

**Drug-induced apoptosis assay**

Cells were treated with different concentrations of cisplatin, 5-FU, or sorafenib for 48 hours. Cells were then collected and stained with propidium iodide (PI), and FITC-conjugated Annexin-V provided by the Annexin-V FLUOS Staining Kit (Roche Diagnostics) according to manufacturer’s instructions. Analysis was conducted by flow cytometry on a LSRFortessa Analyzer (BD Biosciences) and FlowJo software.

**AldeRed ALDH Detection Assay**

ALDH activity of HCC cells was examined according to the kit protocol provided (SCR150, Millipore). Briefly, cells were suspended in 1 ml of AldeRed Assay Buffer supplemented with Verapamil. Specific amount of AldeRed 588-A reagent was added to cells in the test tube and mix well. Aspirate 500 ul of cell suspensions to control tube with DEAB reagent. Incubate test and control tube at 37°C for 30 minutes. Centrifuge all tubes and discard supernatant. Resuspend cell pellets in AldeRed Assay Buffer supplemented with Verapamil followed by flow cytometry.

**Three-dimensional (3D) HCC organoids model**

HCC tissues were obtained from patients undergoing hepatectomy or liver transplantation at Queen Mary Hospital, Hong Kong. Informed consent was obtained from all patients before the collection of liver specimens. Specimen collection and all experiments were approved by the Institutional Review Board of the University of Hong Kong / Hospital Authority Hong Kong West Cluster. For organoid cultures, cells were isolated and cultured according to previously reported protocol(20). For transduction of HCC organoids, the overexpression vectors were transfected into 293T/17 cells with 2.5μg PEI/μg plasmid. Virus-containing supernatant was passed through a 0.45µm filter and ultracentrifuged at 15,000g for 2h. Organoids were first dissociated into single cells with TrypLE (Invitrogen) prior to infection with the lentivirus and then selected with puromycin at 2μg/mL. Note that our organoids have been thoroughly characterized at both molecular and phenotypic levels (Stephanie Ma, unpublished data) and have been used for other HCC studies (Chan LH et al. Cell Reports 2018, Tong M et al. Journal of Hepatology 2018).

**Cell viability assay**

Organoids were treated with indicated concentrations of therapeutic drug sorafenib for several days. Cell viability was measured by CellTiter-Glo assay (Promega) with data presented as percentage of viability relative to blank or vehicle control.

***In vivo* xenograft experiments**

Mice study was performed in accordance with the Committee of the Use of Live Animals in Teaching and Research at the University of Hong Kong. The sample size estimation was conducted by power analysis. For *in vivo* tumorigenic experiment, various numbers of PGC7-transfeced cells were injected subcutaneously into 4-5 week-old NOD/SCID or BALB/c male nude mice. Tumor formation was observed every week. For *in vivo* drug treatment assay, subcutaneous xenografts in 5-week-old BALB/c male nude mice were established with MHCC-97H cells (4×10^6^). Once the tumors reached a size of approximately 4mm in diameter, the mice were randomly divided into four groups by size matching the tumors in all groups (=looking for the distribution that gave the closest tumoral volume’s mean and variance among all groups), each consisting of 6 mice: the vehicle control group, the GANT61 receiving group, the sorafenib receiving group and the combined treatment group. Sorafenib (Selleck.cn) was dissolved in DMSO and further diluted in Kolliphor® EL (Sigma Aldrich) for oral gavage daily; GANT61 (Selleck.cn) was dissolved in solvent (corn oil:ethanol, 4:1) for treatment every other day by intraperitoneal injection. Investigators were blinded for mice treatment group when measuring tumor volume. Tumor volume and body weight were measured every second day. Tumor volume was calculated by 0.5 × *l* × *w^2^*, in which *l* is the length and *w* is the width of the tumor.

**Library construction and whole genome methylation sequencing**

Genomic DNAs were fragmented into 200-300 bp by using Covaris S220; The fragmented DNAs were end repaired and a single ‘A’ nucleotide was appended to the 3’ end of each fragment. After ligating the DNAs to the sequencing adapters, the genomic fragments were bisulfite converted via a EZ DNA Methylation Gold Kit (Zymo Research). The converted DNA fragments were PCR amplified and sequenced as paired-end reads using the Illumina HiSeq™ 4000 platform by the Novogene company (Guangzhou, China).

**Methylation level analysis**

The Raw reads generated were filtered to get high-quality reads using Trimmomatic software. Then the clean reads were mapped to the human reference genome (<ftp://ftp.ensembl.org/pub/release-84/fasta/homo_sapiens/dna/>) using BSMAP software. We used Bismark (Krueger, 2011) to call methylated cytosine and calculate its methylation level based on the following ratio: (mC)/(mC + non-mC). This was calculated for the whole human genome, every chromosome, and for all genomic regions for each of the three methylation contexts (CG, CHG and CHH). The methylation profile across all genomic features is plotted based on the average methylation level for each 100-bp interval. ANOVA analysis was applied to test the significance of the average methylation levels between Vec- and PGC7- transfected cells.

**Analysis of DMRs**:

We identified DMRs (Differentially Methylated Regions) for each cytosine context (CG, CHG, CHH) using swDMR package with the following procedure: (1) a sliding window approach is used to scan the genome with a window length of 1000bp and a step length of 100bp; (2) the region contains at least 10 cytosine sites with more than ten reads coverage per cytosine; (3) average methylation level difference of the region between control group and PGC7 transfected group is larger than 10%; (4) Fisher’s exact test *P* < 0.05 and false discovery rate (FDR) < 0.05; (5) when DMRs are distributed in the promoter region, we define it as DMPs (Differentially Methylated Promoters).

**DNA extraction and bisulfite sequencing**

Genomic DNA was extracted from cell lines by phenol-chloroform method. EpiTECT Bisulfite Kit (Qiagen, Hilden Germany) was used to treat DNA. Bisulfite genomic sequencing was carried out using primers listed in Supplementary Table 1. Genomic DNA extracted was amplified as template by PCR for 35 cycles and then cloned into the pGEM-T Easy vector (Promega) and sequenced as individual clones.

**Supplementary Figure Legends**

**Supplementary Fig. 1. PGC7 is highly expressed in pluripotent stem cells and liver progenitors. (a)** STEM (Short Time-series Expression Miner)-based trend analysis revealed 13 gene expression patterns significantly enriched from the model of *in vitro* hepatocyte differentiation. **(b)** Gene ontology and KEGG pathway enrichment analysis of profile 31 and 28. **(c)** The expression of PGC7, Gli1, Sox2, Nanog and Oct3/4 were analyzed in both undifferentiated ES/iPSCs and differentiated endoderm from GEO database (GSE27087). Undiff, undifferentiated status; ES, embryonic stem cells; iPSCs, induced pluripotent stem cells. **(d)** The association of PGC7 with EpCam, SOX9, CD90 and CD105 in HCC clinical specimens was detected by qRT-PCR. Pearson coefficient R was used to denote the expression correlation. **(e)** Immunofluorescence staining of PGC7 and CK19 in HCC clinical specimens. **(f)** PCR analysis of PGC7 expression in CD133+ and CD133- cells sorted from Huh7 (left panel) or Hep3B (right panel) cell line. ß-actin was used as endogenous control. **(g)** qRT-PCR was conducted to check the expression of PGC7 in spheres and non-spheres formed by MIHA and 97H cells. The expression was calculated as the fold change relative to expression level in non-spheres of 97H cells. Student’s *t*-test was used for statistical analysis, ^*^*P* < 0.05, ^**^*P* < 0.01, data are shown as mean ± SD. Data represent at least three independent experiment. **(h)** Representative dual-color immunofluorescence analysis of cryosectioned spheroids generated from SNU475 cells showing the co-localization of PGC7 (green) and CD133 (red). Three independent experiments were conducted.

**Supplementary Fig. 2. PGC7 silencing suppressed tumor lineage reversion in HCC. (a)** Overexpression of PGC7 was localized in the nuclear of liver or HCC cell lines by immunofluorescent staining. **(b)** Relative expression of PGC7 was detected by qRT-PCR in 8024-shNTC, sh2, sh3 cells. **(c)** PGC7 silencing impaired sphere formation activity in PLC-8024 cells. Calculated ratios were shown in the right panel. **(d)** Stemness-related markers were detected by qRT-PCR in spheres and non-spheres formed by MIHA cells. **(e)** Western Blot was used to detect the expression of SOX2, NANOG and OCT4 in Vec- or PGC7- transfected MIHA cells. ß-actin was used as the loading control. **(f)** Cell viability between shNTC- and sh2-, sh3- transfected PLC8024 cells was compared by XTT assay after treatment with sorafenib at indicated concentrations for 48 hours. **(g)** Apoptotic indexes between shNTC- and sh2-, sh3- transfected cells were compared by flow cytometry with Annexin-V-fluorescein isothiocyanate double staining after treatment with 16 μM sorafenib for 24 hours. The apoptotic index was defined as the percentage of apoptotic cells. **(h)** Vec- or PGC7- transfected 97H cells were injected subcutaneously into the left and the right dorsal flank of nude mice, respectively. Mice with established subcutaneous tumors of similar size were randomly divided into two groups and were given vehicle control or 10 mg/kg sorafenib via oral gavage daily. The tumor size ratio was calculated as the tumor volume in sorafenib group divided by that in vehicle control group. The average tumor volume was expressed as the mean ± SD of 6 mice. **(i)** PLC8024 cells transfected with the empty vector (8024-shNTC) or two short hairpin RNAs targeting PGC7 (8024-sh2/3) were injected subcutaneously into the left and the right dorsal flank of nude mice, respectively. Tumor sizes were observed every week. **(j)** The expression of PGC7, AFP and CK19 were performed by IHC staining in xenograft tumors induced by Vec- or PGC7- transfected 97H cells. Statistics: in Fig. b, c and f-h, Student’s *t*-test was used for statistical analysis, ^*^*P* < 0.05, ^***^*P* < .001. Data represent at least three independent experiments and are shown as mean ± SD.

**Supplementary Fig. 3. Analysis of whole genome methylation sequencing and differentially methylated promoters. (a)** The number of uniquely mapped WGBS reads for each chromosome of Vec- and PGC7- transfected MIHA cells. **(b)** The percent of cytosines in the genome that is covered by differing minimum number of read depths. **(c)** The number and percentage of methylcytosines identified for Vec- and PGC7- transfected MIHA cells in each sequence context. **(d)** Enrichment analysis using Metascape (<https://metascape.org/gp/index.html#/main/step1>) revealed that the DMPs formed interactive network characterized by different clusters. **(e)** Significantly enriched clusters were ranked by *P* value and annotated in different colors.

**Supplementary Fig. 4. PGC7 silencing decreased the expression of GLI1 and MYCN**. **(a)** qRT-PCR was applied in PLC8024 cells transfected with two shRNAs targeting PGC7. **(b)** Relative expression of GLI1 and MYCN were detected by qRT-PCR in PLC8024 cells transfected with empty vector (shCTL) or shRNAs targeting GLI1 (shGLI1). Student’s *t*-test was used for statistical analysis, ^*^*P* < 0.05, ^**^*P* < 0.01, ^***^*P* < .001. Data represent at least three independent experiments and are shown as mean ± SD.

**Supplementary Fig. 5. MYCN inhibitor JQ1 impaired the function of PGC7 on tumor lineage reversion both *in vitro* and *in vivo*. (a,b)** The expression of GLI1 and MYCN were detected in PLC8024 cells by qRT-PCR with the treatment of 10μM GANT61 for 48 hours **(a)** or 6 μM JQ1 for 72 hours **(b)**. **(c)** PLC8024 cells were treated with 6 μM JQ1 for 3 days followed by sphere formation assay. Calculated ratios were shown in the right panel**.** **(d)** Mice body weight were observed every second day and no significant difference was observed during the drug treatment. **(e)** PGC7-overexpressed PLC8024 cells were treated with vehicle control, sorafenib (16μM), JQ1 (10μM), or the combined treatment of both drugs for 2 days, followed by apoptotic index detection. **(f)** Mice with established subcutaneous HCC tumors of similar size were randomly divided into four groups and were given vehicle control, 10 mg/kg sorafenib via oral gavage, 25mg/kg JQ1 via intraperitoneal injection, or combined treatment. Sorafenib was given daily and JQ1 was administered every other day. The average tumor volume was expressed as the mean ± SD of 6 mice. **(g)** The tumors at the end of treatment (left panel) and graph showing the weight of tumors at the end of treatment (right panel). Each dot represents a single tumor. Statistics: in Fig. 5a-c and e-g, Student’s *t*-test was used for statistical analysis, ^*^*P* < 0.05, ^**^*P* < 0.01, ^***^*P* < .001, data are shown as mean ± SD. Data represent at least three independent experiment.

**Supplementary Fig. 6. PGC7 interacts with UHRF1 to induce GLI1 promoter demethylation. (a)** PGC7 was overexpressed in 97H cells. DNMT1 and UHRF2 were immunoprecipitated with anti-DNMT1 or anti-UHRF2 antibodies. The immunoprecipitates were analyzed by western blotting. Three independent experiments were conducted. **(b)** Cell cycle analysis of VEC-, PGC7- transfected 97H and MIHA cells. **(c,d)** Representative images of immunofluorescence staining of UHRF1 in VEC-, PGC7- transfected MIHA and 97H cells **(c)**, as well as in shNTC-, sh2-, sh3- transfected PLC8024 cells **(d)**. Three independent experiments were conducted. **(e)** Quantification of methylation level in each CpG site within the promoter region of GLI1 in MIHA and 97H cells. **(f,g)** The promoter region of GLI1 was analyzed and the methylation status of CpG dinucleotides in cells transfected with shNTC- and sh2/3 was detected by bisulfite genomic sequencing (BGS). The percentage of methylation at each CpG site was displayed in the pie charts **(f)**. Average methylation level (left pane) and quantification in each CpG site (right panel) was depicted **(g)**. **(h)** Sphere formation assay was performed in PGC7-overexpressed 97H cells with or without UHRF1 ectopic expression. Calculated ratios were shown in the right panel. **(i)** The tumors induced by PGC7-overexpressed 97H cells with or without UHRF1 ectopic expression (upper panel), and the graph showing tumor weight and tumor size at the end of observation (lower panel). Each dot point represents a single tumor. **(j)** A schematic diagram showing the proposed working model of the interaction between PGC7, UHRF1 and DNMT1, illustrating how their interaction regulate GLI1/MYCN expression and HCC lineage reversion. Statistics: in Fig. h and i, Student’s *t*-test was used for statistical analysis, ^**^*P* < 0.01, data are shown as mean ± SD.

**Supplementary Fig. 7. Double-immunofluorescence staining of MYCN and GLI1 in xenograft tumors induced by 97H-PGC7 cells.**
