# Supplementary figures and images for: PGC7 promotes tumor oncogenic dedifferentiation through remodeling DNA methylation pattern for key developmental transcription factors
Source: Cell Death Differ. 2021 Jan 26;28(6):1955–70. doi: 10.1038/s41418-020-00726-3 (PMC8185079; doi:10.1038/s41418-020-00726-3)

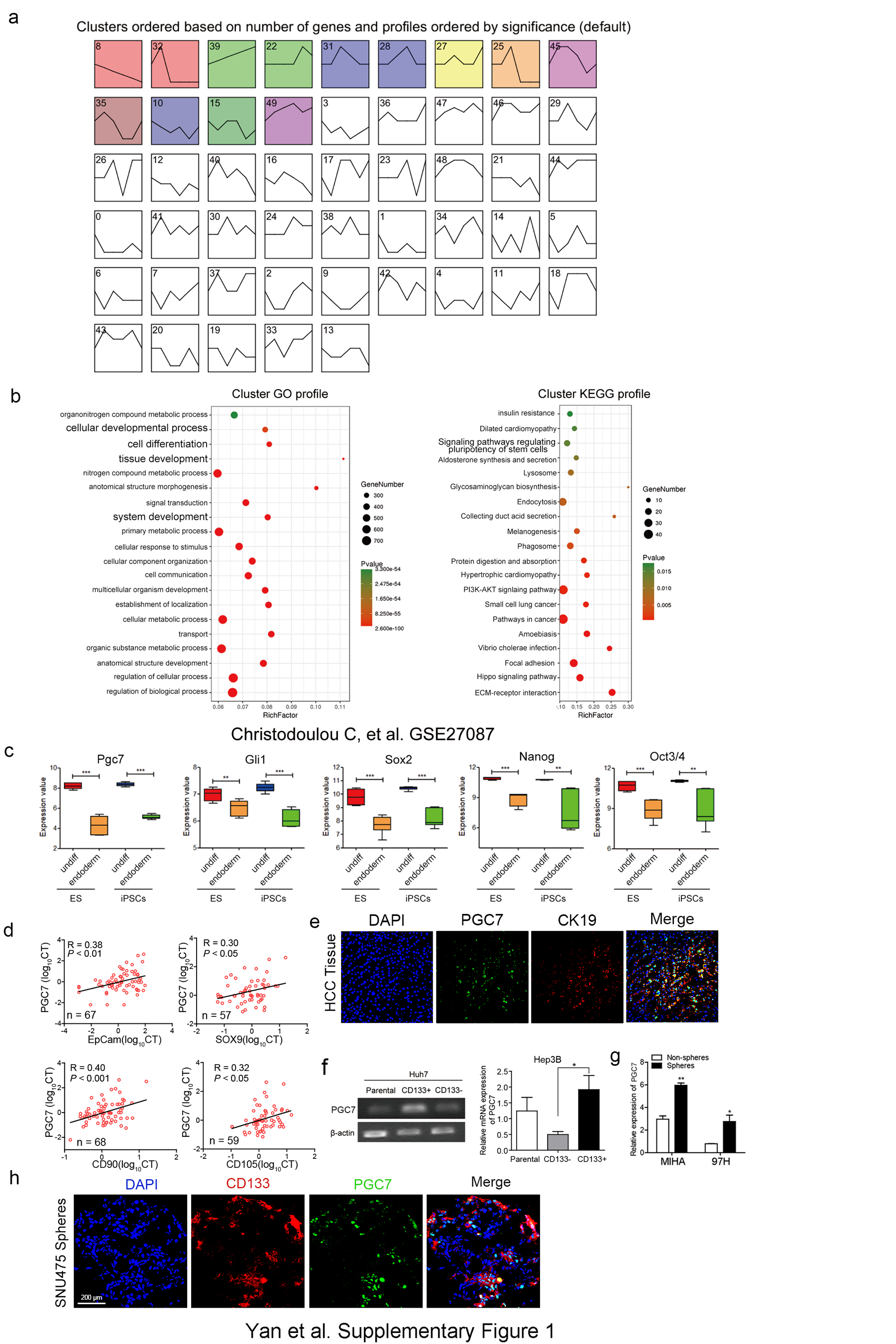

Supplement: Supplementary file 2 — Supplementary Figure 1 [file 41418_2020_726_MOESM2_ESM.tif]

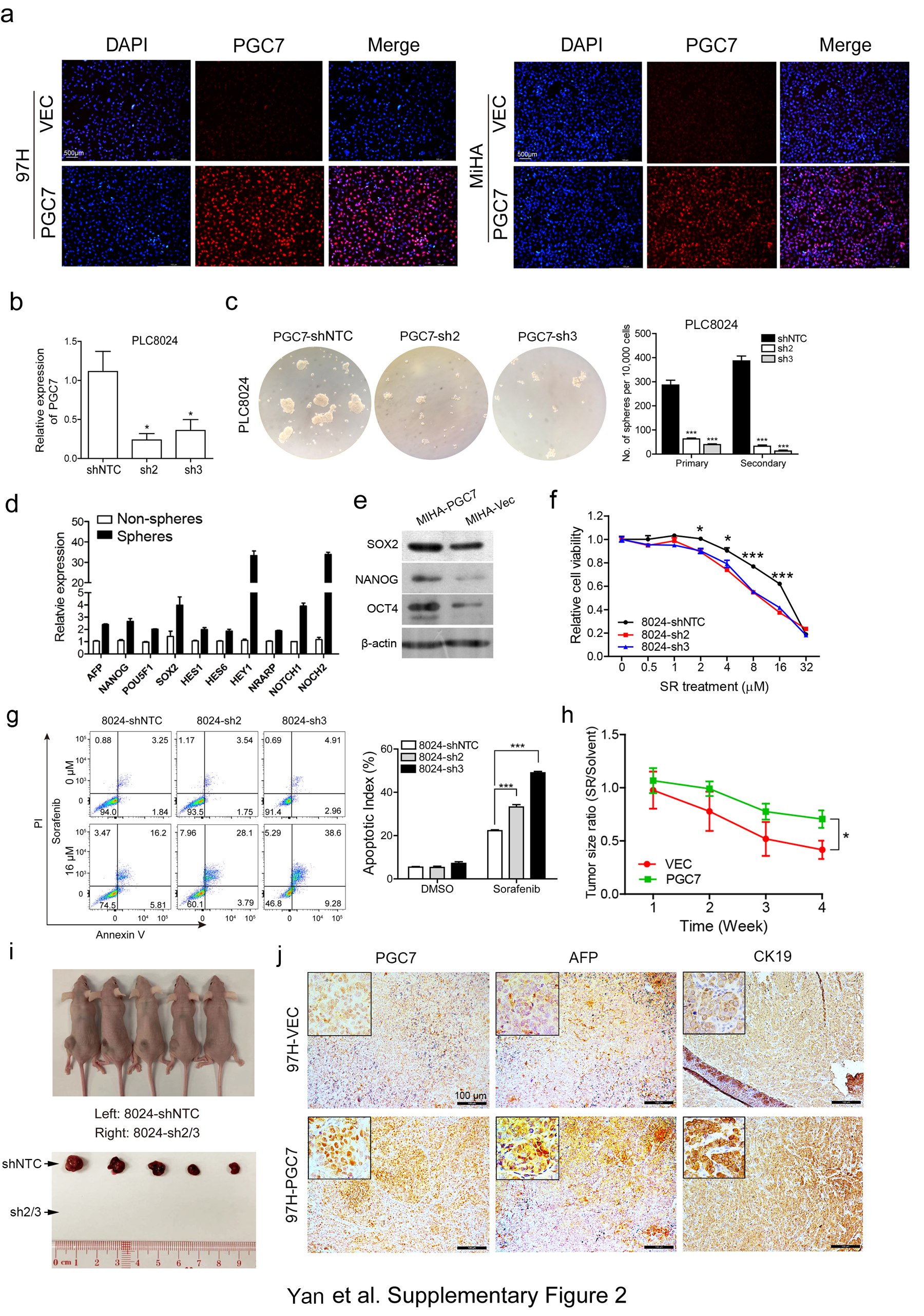

Supplement: Supplementary file 3 — Supplementary Figure 2 [file 41418_2020_726_MOESM3_ESM.tif]

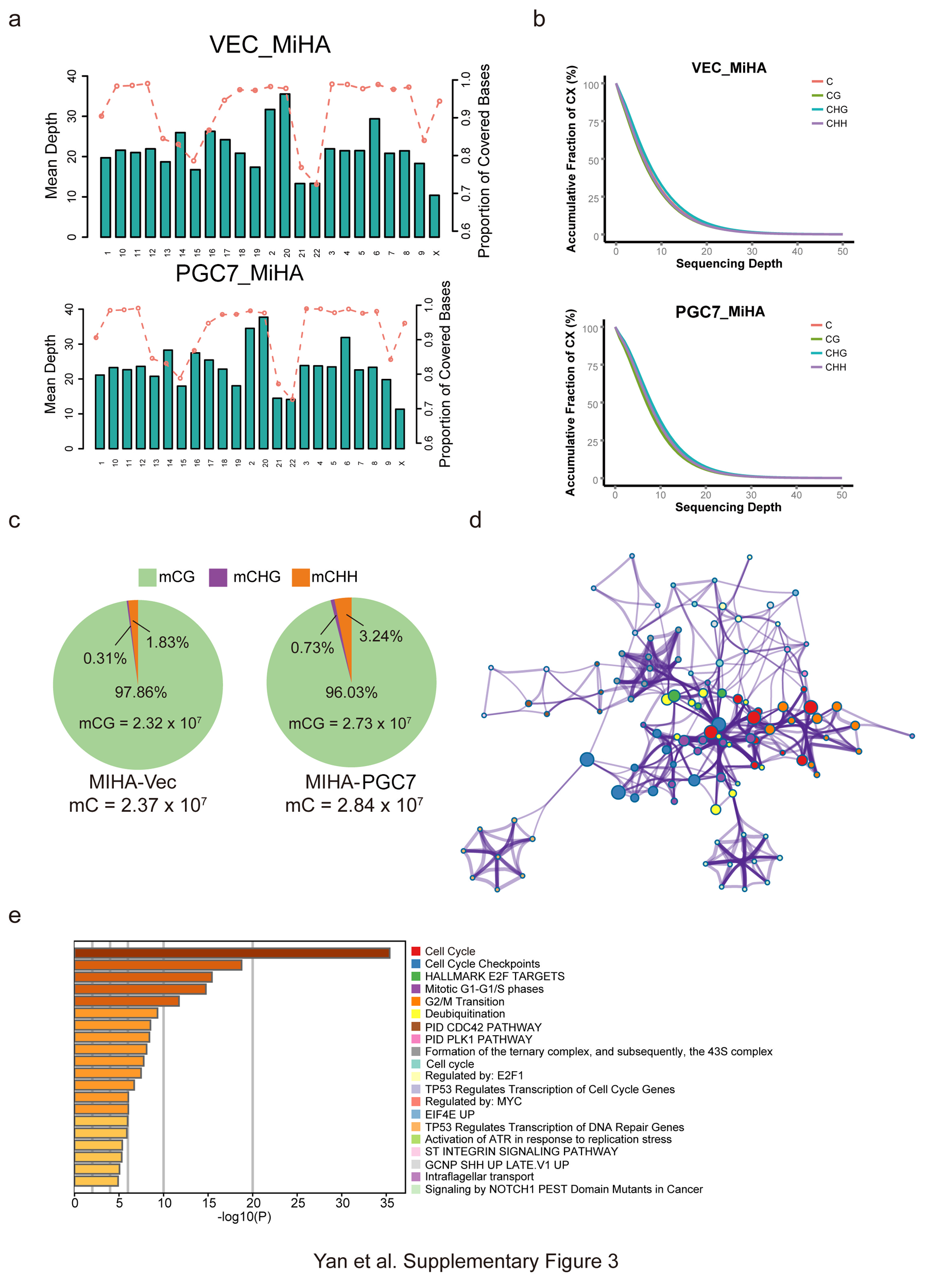

Supplement: Supplementary file 4 — Supplementary Figure 3 [file 41418_2020_726_MOESM4_ESM.tif]

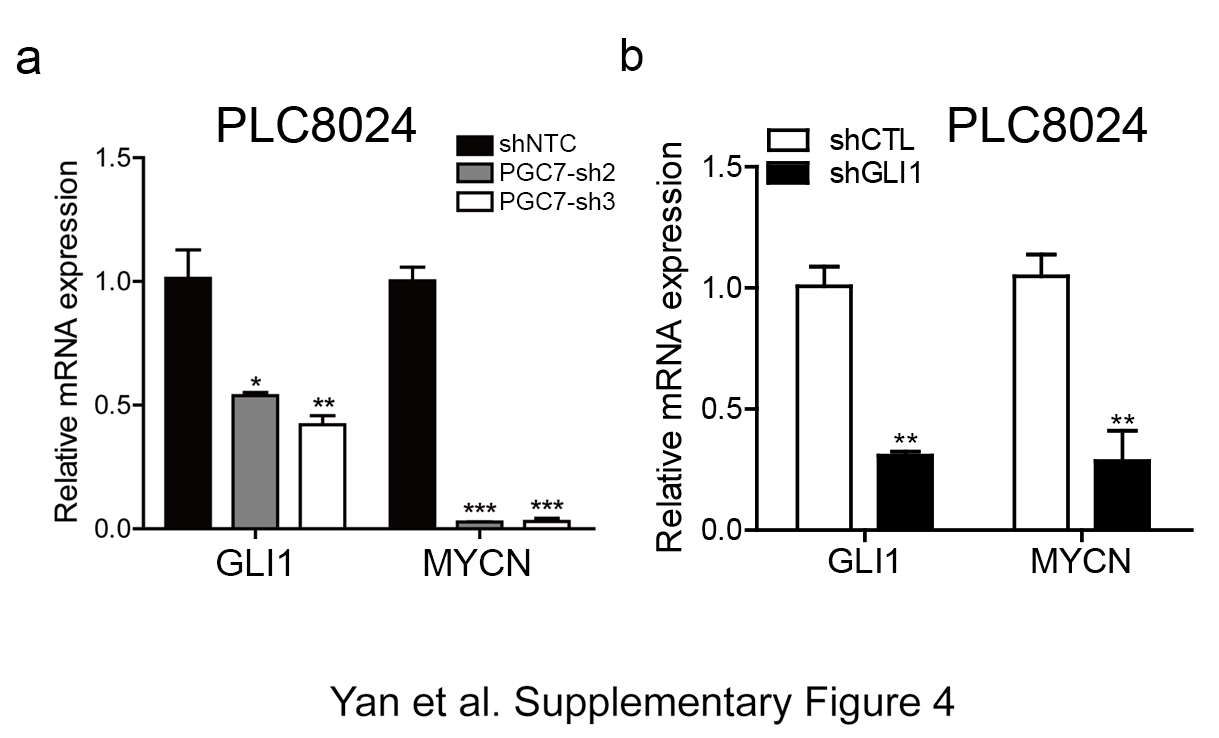

Supplement: Supplementary file 5 — Supplementary Figure 4 [file 41418_2020_726_MOESM5_ESM.tif]

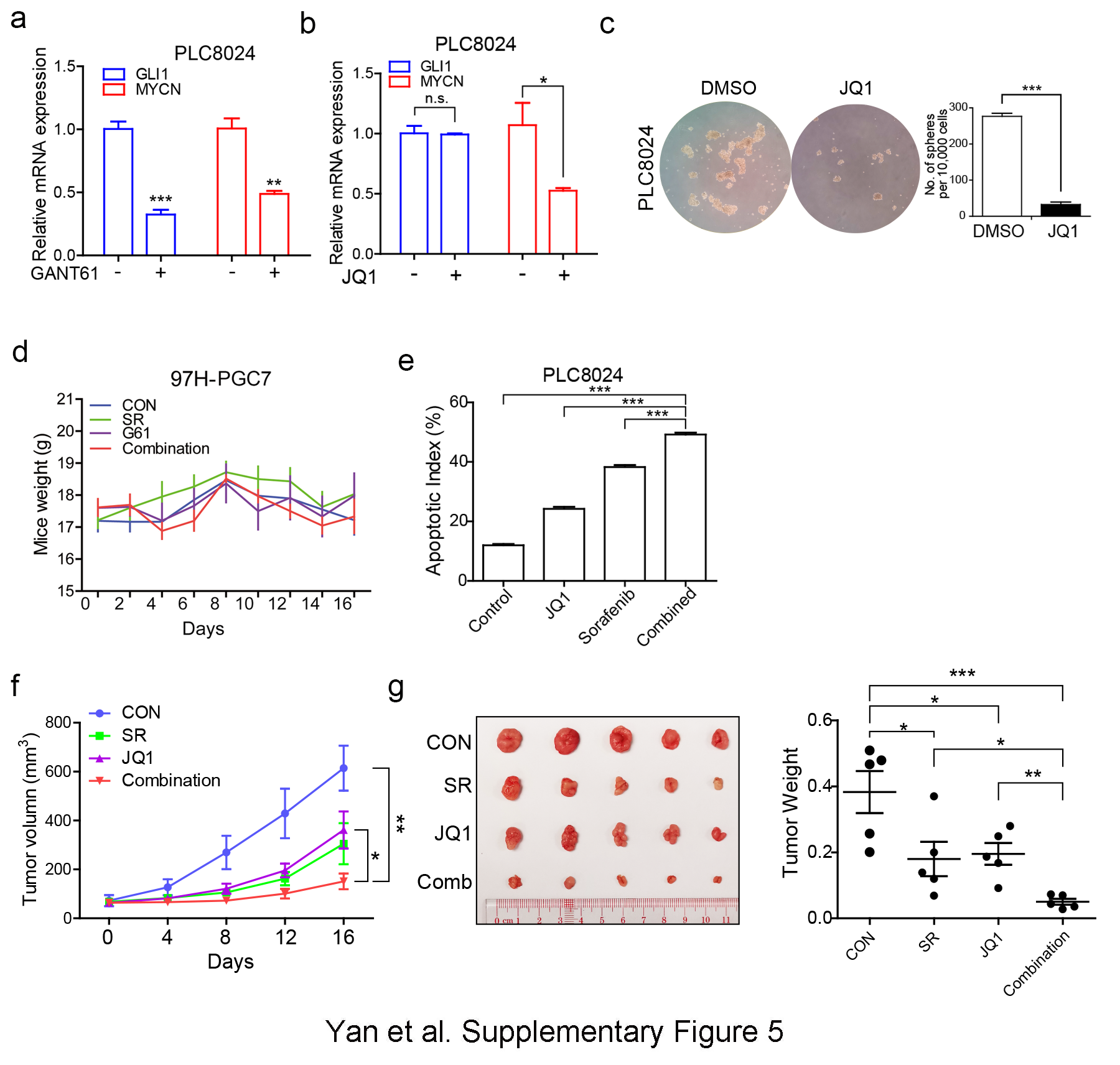

Supplement: Supplementary file 6 — Supplementary Figure 5 [file 41418_2020_726_MOESM6_ESM.tif]

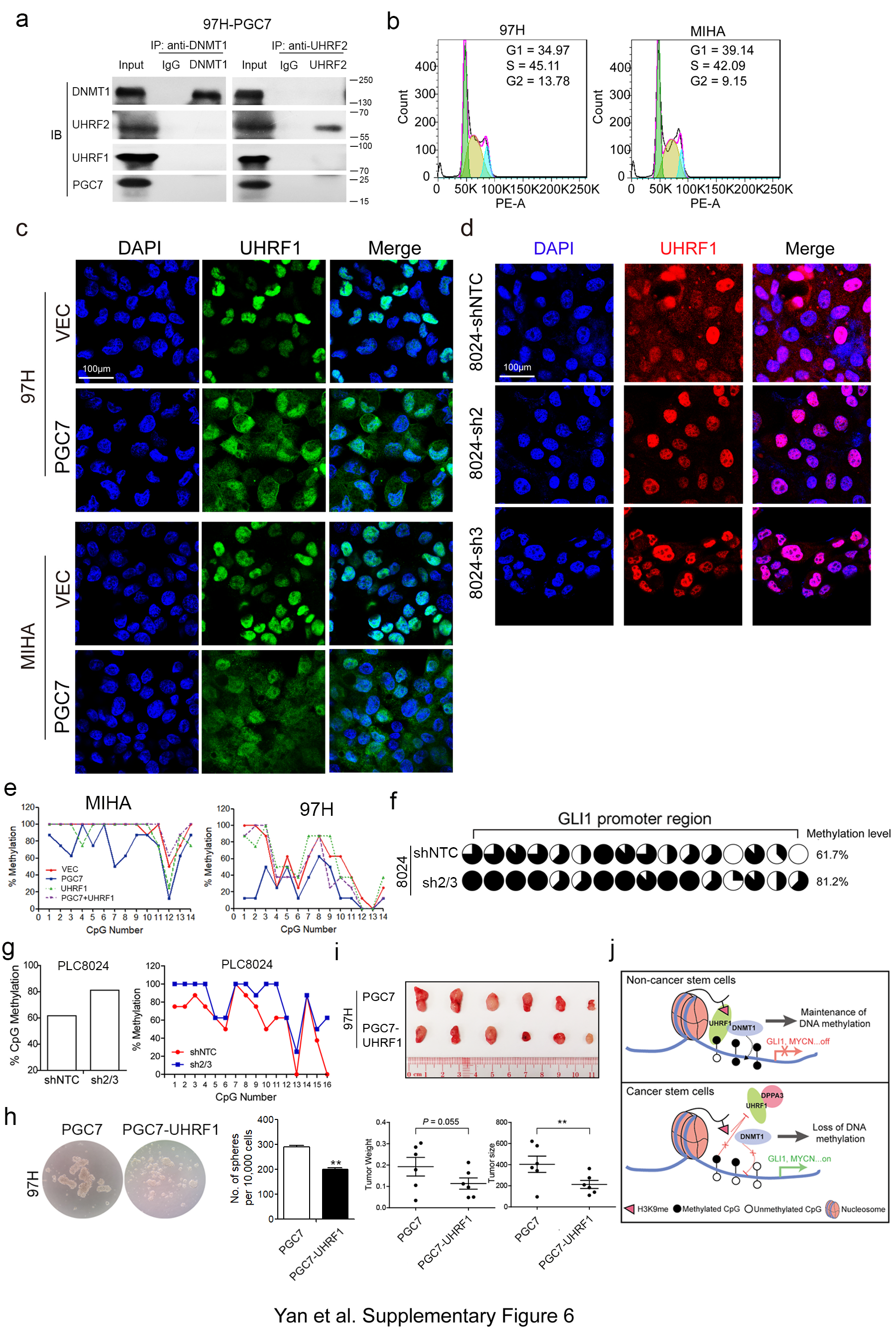

Supplement: Supplementary file 7 — Supplementary Figure 6 [file 41418_2020_726_MOESM7_ESM.tif]

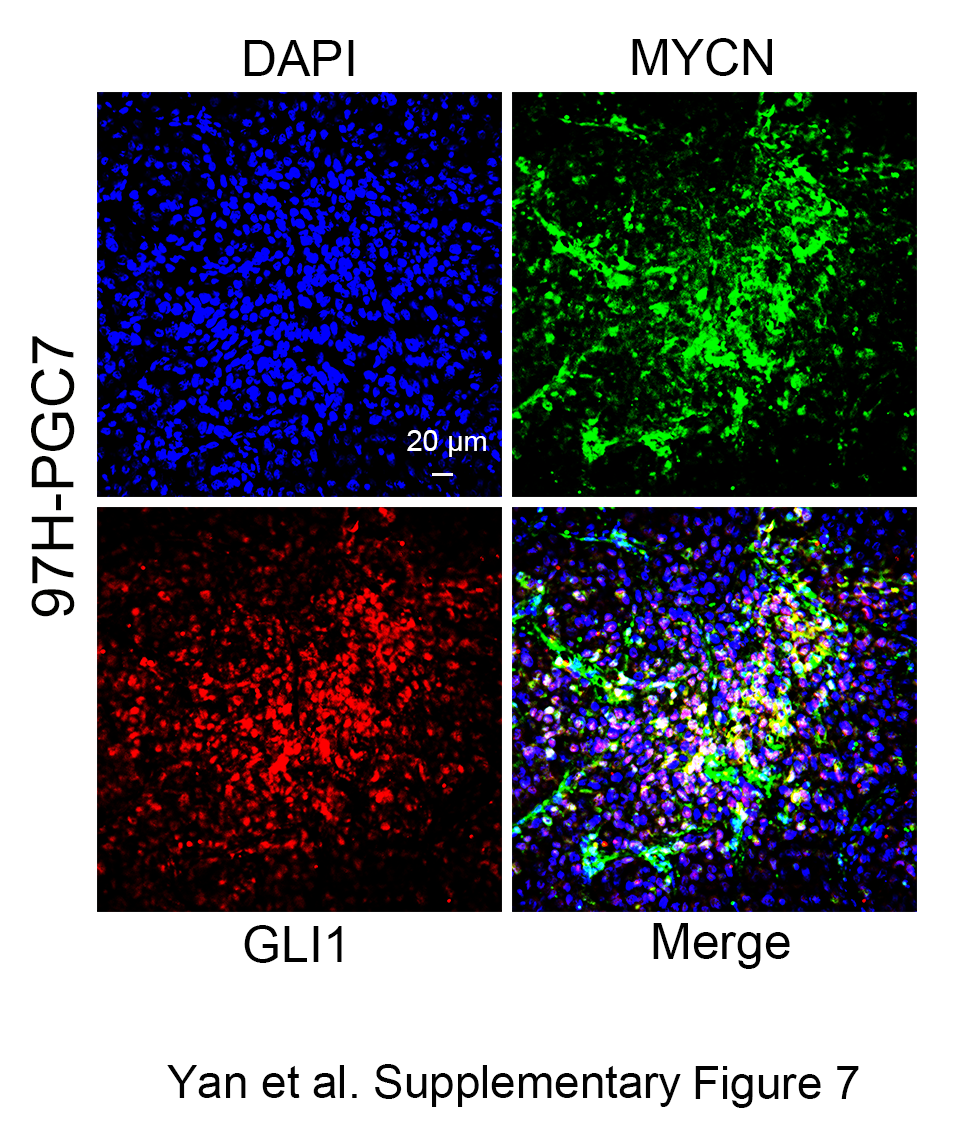

Supplement: Supplementary file 8 — Supplementary Figure 7 [file 41418_2020_726_MOESM8_ESM.tif]
