# Supplementary material for: PGC7 promotes tumor oncogenic dedifferentiation through remodeling DNA methylation pattern for key developmental transcription factors
Source: Cell Death Differ. 2021 Jan 26;28(6):1955–70. doi: 10.1038/s41418-020-00726-3 (PMC8185079; doi:10.1038/s41418-020-00726-3)
Supplement: Supplementary file 9 — Supplementary table 1 [file 41418_2020_726_MOESM9_ESM.docx]

**Supplementary Table 1.** **Sequences of primers used in qPCR and bisulfite genomic sequencing.**

| Primer | Sequence (5’-3’) |
| --- | --- |
| qRT-PGC7-F | TAGCGAATCTGTTTCCCCTCT |
| qRT- PGC7-R | CTGCTGTAAAGCCACTCATCTT |
| qRT-CD133-F | GGCCCAGTACAACACTACCAA |
| qRT- CD133-R | ATTCCGCCTCCTAGCACTGAA |
| qRT-18S-F | AACCCGTTGAACCCCATT |
| qRT-18S-R | CCATCCAATCGGTAGTAGCG |
| qRT-CK19 -F | ACCAAGTTTGAGACGGAACAG |
| qRT-CK19-R | CCCTCAGCGTACTGATTTCCT |
| qRT-ALB-F | GAGACCAGAGGTTGATGTGATG |
| qRT-ALB-R | AGTTCCGGGGCATAAAAGTAAG |
| qRT-G6PC-F | ACTGGCTCAACCTCGTCTTTA |
| qRT-G6PC-R | CGGAAGTGTTGCTGTAGTAGTCA |
| qRT-MYCN-F | TGATCCTCAAACGATGCCTTC |
| qRT-MYCN-R | GGACGCCTCGCTCTTTATCT |
| qRT-GLI1-F | GGGTGCCGGAAGTCATACTC |
| qRT-GLI1-R | GCTAGGATCTGTATAGCGTTTGG |
| qRT-KCNQ4-F | CTGTCCACTATCCAGGAGCAC |
| qRT-KCNQ4-R | CGAAAACCACGATCATCACGAA |
| qRT-NFATC1-F | TGTGCCGGAATCCTGAAACTC |
| qRT-NFATC1-R | GAGCATTCGATGGGGTTGGAG |
| qRT-MYT1L-F | CCTGCTCCTAAACGAAAGCCA |
| qRT-MYT1L-R | TCCCATCACTGTCGTCACACT |
| qRT-MACROD2-F | AGATGACCTTAGAAGAGAGACGC |
| qRT-MACROD2-R | CCATGATAGAATGCTGTTCAGGG |
| qRT-FBXO24-F | ACACGAAGGGCCTGTATTTCC |
| qRT-FBXO24-R | CCCACGTAGTCAAGAATGAAGAC |
| qRT-CGREF1-F | TGCCCTCCATGACTATGACCA |
| qRT-CGREF1-R | GGTAGGAGAGTTGGCAGCTC |
| qRT-RNF222-F | ATGACTGCCTGGTCAAGTACC |
| qRT-RNF222-R | CTTGCTGAGGAATGTGACGTAG |
| qRT-ZNF222-F | TTGACTTCCACCATAGAACCCA |
| qRT-ZNF222-R | GACCGGCATACAAGCCTCTT |
|  |  |

| Primer | Sequence (5’-3’) |
| --- | --- |
| qRT-BCL11A-F | CGCCAGAGGATGACGATTGTT |
| qRT-BCL11A-R | CCAGGCGTGGGGATTAGAG |
| qRT-MLPH-F | AAGCCCGCTTCAAGAGGTTC |
| qRT-MLPH-R | TGGTCGCTGTCTCCACTTCT |
| qRT-STMN4-F | CCTGAATTGGTGCGTCATTTCC |
| qRT-STMN4-R | TCCTCAGCCGCTTCTAGTTTC |
| qRT-RASA4-F | CAGCCGGGACGACGTTATC |
| qRT-RASA4-R | CCACCCGCTGAAACCCTTAG |
| qRT-DENND2A-F | CGGACAGGAGGATTATCTGCC |
| qRT-DENND2A-R | CTGGCTGGCTTAGGTCTTGC |
| qRT-GC-F | GAGGGCCTGGAACGAAAGC |
| qRT-GC-R | AGCAGGACCCTACCATAGAAAG |
| BGS-GLI1-F1 | ATGTGGTGAATTGATAGATGTAAATTATATG |
| BGS-GLI1-R1 | CAATAACTCAAACCTATAATCCCAACAC |
| BGS-GLI1-F2 | GGGTGGGGAGAAGAAAGTAAGGT |
| BGS-GLI1-R2 | ACCCCACACATACTCCTATCC |
| BGS-GLI1-F3 | GGAGGTTTGTTTGTGGGGTTG |
| BGS-GLI1-R3 | ACTCTTCCAAACTTTCTACTCCCT |
|  |  |
